# Pioneering insights into the diving behavior of early-stage sea turtles revealed by novel marine miniaturized satellite tags

**DOI:** 10.1038/s41598-026-47239-6

**Published:** 2026-04-09

**Authors:** Tony Candela, Philippe Gaspar, Helen Bailey, Jeanette Wyneken, Emily Turla, Talitha Noble-Trull, Ronel Nel, Hirun Kanghae, Pinsak Suraswadi, Tipamat Upanoi, Junichi Okuyama, Isao Kawazu, Ken Maeda, Kaho Mizuochi, Nene Ogino, Frederic Vandeperre, Ana Mafalda Sousa, Andrea Herguedas, Mark De Boer, Florence Dell’Amico, Antonieta Nunes, Joao Neves, Isabel Gaspar, George L. Shillinger

**Affiliations:** 1 Upwell, Monterey, CA USA; 2 Mercator Ocean International, Toulouse, France; 3 Aquarium La Rochelle, Centre d’Etudes et de Soins pour les Tortues Marines, La Rochelle, France; 4 Blue Wave Consulting, LLC, Baltimore, MD USA; 5 FAU Marine Science Laboratory, Department of Biological Sciences, Florida Atlantic University, Boca Raton, FL USA; 6 Two Oceans Aquarium Foundation, Cape Town, South Africa; 7 Nelson Mandela University, Port Elizabeth, South Africa; 8 Department of Marine and Coastal Resources, Phuket Marine Biological Center, Phuket, Thailand; 9 Department of Marine and Coastal Resources, Bangkok, Thailand; 10 Marine and Coastal Resources Research Center (Upper Andaman Sea), Phuket, Thailand; 11 Sea Turtle Ecology Lab, Yokohama, Kanagawa Japan; 12 Okinawa Churashima Foundation, Motobu, Okinawa Japan; 13 Okinawa Churaumi Aquarium, Motobu, Okinawa Japan; 14 Institute of Marine Sciences, IICM Okeanos, University of the Azores, 9901-862 Horta, Portugal; 15 Institute of Marine Research, IMAR, 9900-138 Horta, Portugal; 16 Rotterdam Zoo, Rotterdam, The Netherlands; 17 Zoomarine Algarve, Mundo Aquatico SA, Guia, Albufeira, Portugal

**Keywords:** Juvenile sea turtles, Vertical behavior, Diving activity, Miniaturized satellite tags, Foraging strategy, Migration, Ecology, Ecology, Ocean sciences

## Abstract

Understanding how sea turtles interact with their environment during their early oceanic life stages—the time often termed “Lost Years”—is critical for informing conservation strategies. This study provides the most comprehensive analysis to date of vertical diving behavior in early life stages of two sea turtles species: leatherback (*Dermochelys coriacea*) and loggerhead (*Caretta caretta*) sea turtles. Using over 2400 daily records from 71 individuals equipped with miniaturized satellite tags across multiple ocean basins, analyses revealed a clear ontogenetic progression. As a turtle’s size increases, they dive deeper and longer, with increasingly structured use of the water column. Some individuals also exhibited brief acclimatization phases after release, gradually adopting deeper and more consistent diving patterns. Environmental analyses highlight qualitative associations between vertical behavior in larger turtles and the structure of the water column, including thermal gradients and subsurface layers potentially associated with prey availability. These findings offer novel insights into the development, acclimatization, and ecological drivers of vertical movement in “Lost Years” sea turtles, helping to bridge knowledge gaps during this critical life stage.

**Supplementary Information:**

The online version contains supplementary material available at 10.1038/s41598-026-47239-6.

## Introduction

 To effectively manage and protect a species, knowledge of its spatial distribution through all its life stages is essential^1,2^. Key habitats and areas where threats are most likely to occur must be identified to inform the development of appropriate conservation strategies. Acquiring such knowledge for endangered or threatened highly migratory marine species such as sea turtles is a major challenge^3^ since observations are especially difficult to obtain in the open ocean and even more so for very young individuals due to their small size. Survivorship of individuals during their juvenile life stages is particularly important for population-wide persistence^4,5^. However, their ecology remains enigmatic from the moment they crawl into the sea until they reappear, many years later, as large juveniles or adults in neritic foraging areas or in the direct vicinity of their nesting beaches. This obscure period of life is commonly referred to as the “Lost Years”^6^.

Until recently, the very small body sizes and prolonged dispersal phases of juveniles posed many challenges for the development of satellite tracking studies^1,2,7^. Recent advances in tag miniaturization technologies have enabled researchers to undertake groundbreaking studies on the movements and dispersal of early-stage sea turtles^7–12^, significantly extending tracking durations of very early stage (neonate) turtles acquired through active tracking techniques with miniaturized acoustic tags^13,14^. Novel miniaturized satellite tags (some weighing less than 3 g) equipped with solar cells and pressure sensors have prolonged tracking durations and provided first-ever dive data for early-stage sea turtles for periods ranging from a few days to several months^12^, and in some cases nearly a year or even longer. In addition to horizontal movement data, some miniaturized satellite tags now offer groundbreaking capabilities for collecting long-term, fine-scale observations on how young sea turtles move vertically during their initial dispersal stages. Understanding how these early-stage sea turtles interact with their environment in three dimensions provides an important ecological context to their migratory and survival strategies. Such knowledge of juvenile diving patterns provides a basis for conservation actions, for example by informing the adjustment of fishing gear deployment depth, and the design of marine protected areas that reflect the vertical habitat use of these species^15–19^.

Our study harnesses an unprecedented dataset generated through deployments conducted since 2022 at different locations in the Atlantic, Indian and Pacific oceans. We compiled data from more than a hundred miniaturized satellite tags^12^ equipped on early life stage juvenile loggerhead (*Caretta caretta*, Vulnerable^20^ and leatherback (*Dermochelys coriacea*, Vulnerable^21^ sea turtles, from post-hatchlings with straight carapace length (SCL) < 10 cm to small juveniles (SCL < 40 cm). Our study aims to provide critical insights into the early behavioral development of these species, with a particular focus on their diving behavior. By improving understanding of behavioral mechanisms during the Lost Years, our work provides empirically grounded data that can help improve our capacity to predict juvenile sea turtle movements in the open ocean and identify potential overlaps with anthropogenic threats.

## Materials and methods

### Study animals

**Fig. 1:**
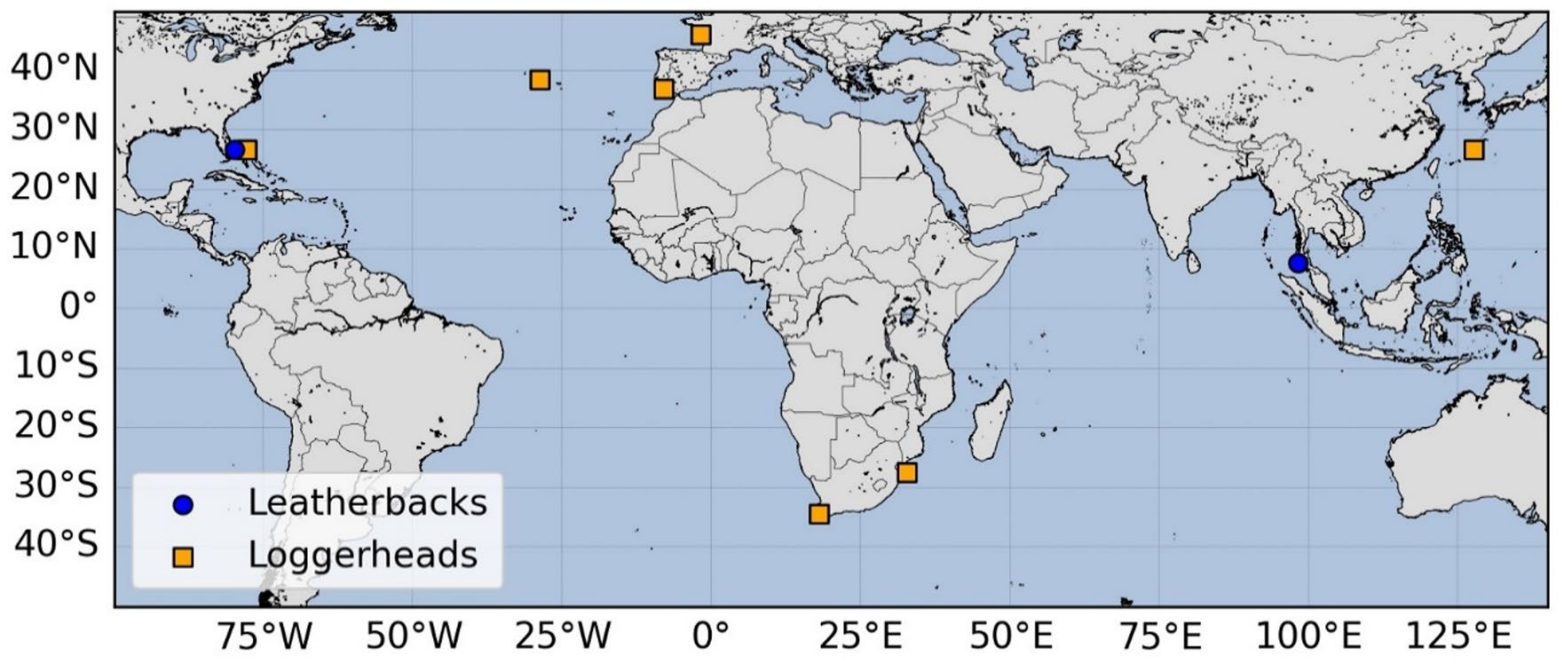
Release locations for leatherback and loggerhead sea turtles. Map with the release location of sea turtles deployed for this study. Leatherbacks (blue circles) were released from two locations: Eastern Florida (USA) and Western Thailand. Loggerheads (orange squares) were released from seven locations: Eastern Florida (USA), the Azores archipelago (Portugal), the Western France, Southern Portugal, Western and Eastern South Africa, and Southern Japan islands.

Our study involved a total of 105 young sea turtles, including both loggerheads and leatherbacks, released across 7 locations worldwide (Fig. 1) between 2022 and 2024. The turtles were either captive-reared, wild-caught, or rehabilitated, and ranged from post-hatchlings with straight carapace length (SCL) < 10 cm to small juveniles (SCL < 40 cm).

Our dataset included 58 loggerheads, 24 of which were captive-reared for approximately 3 months at Florida Atlantic University (Florida, USA) or Churaumi Aquarium (Okinawa, Japan) post-emergence from in situ nests. The remaining 34 loggerheads were either rehabilitated (recovery after stranding or injury), or were wild-caught. The loggerhead SCL ranged from 9.14 to 28.00 cm. The captive-reared individuals released off Eastern Florida, USA (*n* = 13) and off Ie Island, Japan (*n* = 11) were the smallest (SCL from 9.14 to 16.20 cm). Rehabilitated sea turtles released off South Africa (*n* = 22, SCL from 11.90 to 16.20 cm) and in the Bay of Biscay along the Atlantic coast of France (*n* = 5, SCL from 16.40 to 20.40 cm) were slightly larger. Those released within the Azores (*n* = 6) included four small individuals (SCL from 11.20 to 14.40 cm), that were wild-caught using a dip net off the coast, and two large ones (26.00 and 28.00 cm), that were found stranded in the Netherlands and later transported to the Azores for release. The single turtle released off southern Portugal measured 26.60 cm.

The remaining 47 sea turtles in the dataset were all captive-reared leatherbacks. These included a subset of 36 turtles collected post-emergence from in situ nests in Boca Raton, Florida, USA, reared at Florida Atlantic University for about 2 or 3 months and then released in the Gulf Stream off the coast of Eastern Florida. Upon release, the turtles’ SCL ranged from 7.42 to 12.10 cm. The remaining 11 leatherbacks were raised for just over a year (range: 375–377 days) at the Phuket Marine Biological Center^22^ and released from Thailand’s west coast into the Andaman Sea. The SCLs of these individuals ranged from 21.96 to 36.00 cm.

We compared observed size ranges with published growth curves for leatherbacks^23^ and for loggerheads^24^. While leatherback sizes were generally consistent with these growth trajectories, the smallest loggerheads appeared slightly above expected values, which may reflect differences between captive-reared and wild individuals growth patterns. Detailed information for all individuals included in the study is presented in Supplementary material S1.

In the following sections, all the released individuals were classified according to their species, origin (i.e., wild-caught/rehabilitated, or captive-reared), and size class based on SCL at the time of release: small for SCL smaller than 15.00 cm, intermediate to SCL between 15.00 and 21.00 cm or large for SCL larger than 21.00 cm. These size classes were used to reflect different ontogenetic stages.

### Platform transmitter terminals

All the Platform Transmitter Terminals (PTT) used in this study were miniaturized satellite tags designed and manufactured by Lotek Wireless Inc. (Newmarket, Ontario, Canada). In order to conform tag size to body-size standard and mitigate the perturbations^1,25–27^, all PTTs plus attachment materials weighed 5% or less of the turtle’s body mass. All sea turtles included in this study were each equipped with one of two types of tags, based on tag availability, provided that the 5% criterion was satisfied. The smallest tag model was K4H 130B Dive (length × width × height: 26.0 × 14.7 × 7.7 mm, 3.1 g in air). The larger tag model was K4G 132 A Sensor (47.7 × 30.0 × 21.9 mm, 12.1 g in air). All technical information (e.g., data transmission, power supply, sensors) of these PTTs and the attachment methods are available in Candela et al.^12^.

In addition to the Argos locations, the PTTs were expected to provide daily summaries of the previous day’s vertical (surface or dive depth) behavior with 3 distinct variables:

1. the fraction of the day (from 00:00 to 23:59 UTC) spent underwater (%TU) based on the saltwater switch, with the dry state associated with the time spent at the surface and the wet state associated with the time spent underwater;
2. the maximum depth reached during this 24-h period, hereafter referred to as the Maximum Daily Diving Depth (M3D);
3. the Time-at-Depth (TaD) distribution representing the distribution of underwater time (in %) within 7 depth layers. For loggerhead sea turtles, depth bins were 0–5, 5–10, 10–15, 15–20, 20–25, 25–30 and below 30 m. For leatherbacks, depth bins were 0–10, 10–20, 20–30, 30–40, 40–50, 50–60 and below 60 m.

Because the variable accounting for time spent underwater (%TU) is directly derived from the saltwater switch as the fraction of time when the sensor is wet, it does not always clearly distinguish between turtles being truly submerged and those remaining at or near the surface. For instance, short periods of sensor wetness can occur at the surface due to splashing, rain, or water droplets. Conversely, the dry state can be slightly delayed when turtles emerge at the surface. To better isolate actual diving behavior, we defined a new variable, Diving Time (%DT), as the proportion of time spent below the first sampled vertical layer (i.e., below 5 m for loggerheads and 10 m for leatherbacks). This metric, used alongside Maximum Daily Dive Depth (M3D) and the Time-at-Depth (TaD) distribution, provided a more conservative assessment of the diving activity.

To characterize the mean behavior of a group of individuals, we used the mean ± standard deviation (mean ± SD) for continuous variables (e.g., M3D, tracking duration). However, for variables expressed as percentages (e.g., %DT and TaD distributions), this approach was not optimal. Instead, we used the mean with its 95% confidence interval (mean [CI95]), as recommended for proportion data^28^. This method retained information on the group’s average behavior while providing a more appropriate and robust estimate of the precision of the mean.

### Data processing

In order to standardize the dataset, trajectories were resampled with the Move Persistence State-Space Model aniMotum^29^ allowing for regularly time-spaced locations taking into account Argos location’ error ellipses. The resampling interval was set to 12 h to get locations at 00:00 and 12:00 UTC every day.

Subsequently, oceanographic data from Copernicus Marine Service global ocean models^30–32^ were interpolated at the resampled 12:00 UTC daily locations of the turtles using distance-weighted nearest neighbor interpolations. Daily 3D ocean current velocities and 3D sea water temperatures were retrieved from the Global Ocean Physics Analysis and Forecast product^30^ which has a horizontal resolution of 1/12° and 50 vertical levels. Daily Significant Wave Heights (SWH) were retrieved and calculated from hourly data of the Global Ocean Waves Analysis and Forecast product^31,33,34^ with a horizontal resolution of 1/12°. Bathymetry data were retrieved from the General Bathymetric Chart of the Oceans (GEBCO) dataset^35^ which has a horizontal resolution of 15 arc-seconds. Daily 3D concentrations of chlorophyll-a (chl-a) were retrieved from the Global Ocean Biogeochemistry Analysis and Forecast product^32^ with a horizontal resolution of 1/12° and 50 vertical levels. It is used as a proxy for prey abundance.

We fit generalized additive mixed models (GAMMs) to test whether diving behavior varied in relation to time since release or size of the turtle. We fit models for each of the two turtle species with two response variables: the %DT (formatted as a proportion between 0 and 1) with a beta distribution and logit link function, and the M3D with a gamma distribution and log link function. The explanatory smoother variables were the time elapsed since the release (in days) and SCL (in cm) at the time of release. The smoothing functions of these continuous variables were limited to four degrees of freedom to limit overfitting the data. Individual IDs were included as a random effect to account for behavior specific to the individual. Statistical significance was assessed based on p-values, with effects considered significant at p-value < 0.05.

The software R version 4.2.2^36^ was used for trajectory resampling and for statistical (i.e., GAMMs) analyses and associated figures, using respectively the aniMotum^29^ and mgcv^37^ packages. All other processing works, including environmental variable interpolations, analyses, and figure production, were performed in Python version 3.14.1.

### Ethic statements

Work with leatherbacks and loggerheads from Florida, USA, complied with Florida FWC permit MTP-073, and FAU IACUC Protocol A22-30. Work involving loggerheads from South Africa has been conducted under the Ethics Approval TOA-RES 07_25. Work with leatherbacks in Thailand was conducted by the Phuket Marine Biological Center and carried out in accordance with the Thailand Wildlife Conservation and Protection Act, in adherence with Constitutional Law of Thailand. For the loggerheads released in Japan, the entire experimental protocol—including hatchling collection on the beach, captive-rearing, transmitter attachment, and release—has been approved by both the Okinawa Fisheries Coordination Committee (6-k3) and the Okinawa Prefectural Government (6–35). The work with loggerheads in the Azores was conducted under the licenses LMAS-DRPM/2022/06, LMAS-DRPM/2023/15 issued by the Regional Directorate for Maritime Policies and approved by the board responsible for animal welfare of the University of the Azores (Órgão Responsável pelo Bem-Estar dos Animais; COM/ORBEA/2024/002). The two loggerheads that underwent rehabilitation in the Netherlands prior to their release in the Azores were rehabilitated under a license issued by the Omgevingsdienst Haaglanden and registered under Case Number 01004761. For the French turtles, this study meets the legal requirements of the country and the Rescue Center where the work was carried out and adhered to all institutional guidelines. The Prefectoral Order n°2004-1104 and its modifications n°2024 02286 approved the opening of the Centre d’Etudes et de Soins pour les Tortues Marines (CESTM) and delivered to Mrs. Florence Dell’Amico a certificate (n°2017 02173) to conduct care practices on non-domestic animals. The French Ministerial Order (30/12/2020) acts as an exception to the strict protection of species, authorizing the manipulation of protected species when found bycaught, drifting at sea or stranded and for equipping with satellite tags the marine turtles. All wildlife rehabilitation procedures conducted in mainland Portugal were performed under Operational License n°2012 PT/03/CR, issued to the Porto d’Abrigo Rehabilitation Center at Zoomarine Portugal, in full compliance with the national regulations governing the rehabilitation of wild fauna.

## Results

### Tracking durations and filtering process

**Fig. 2:**
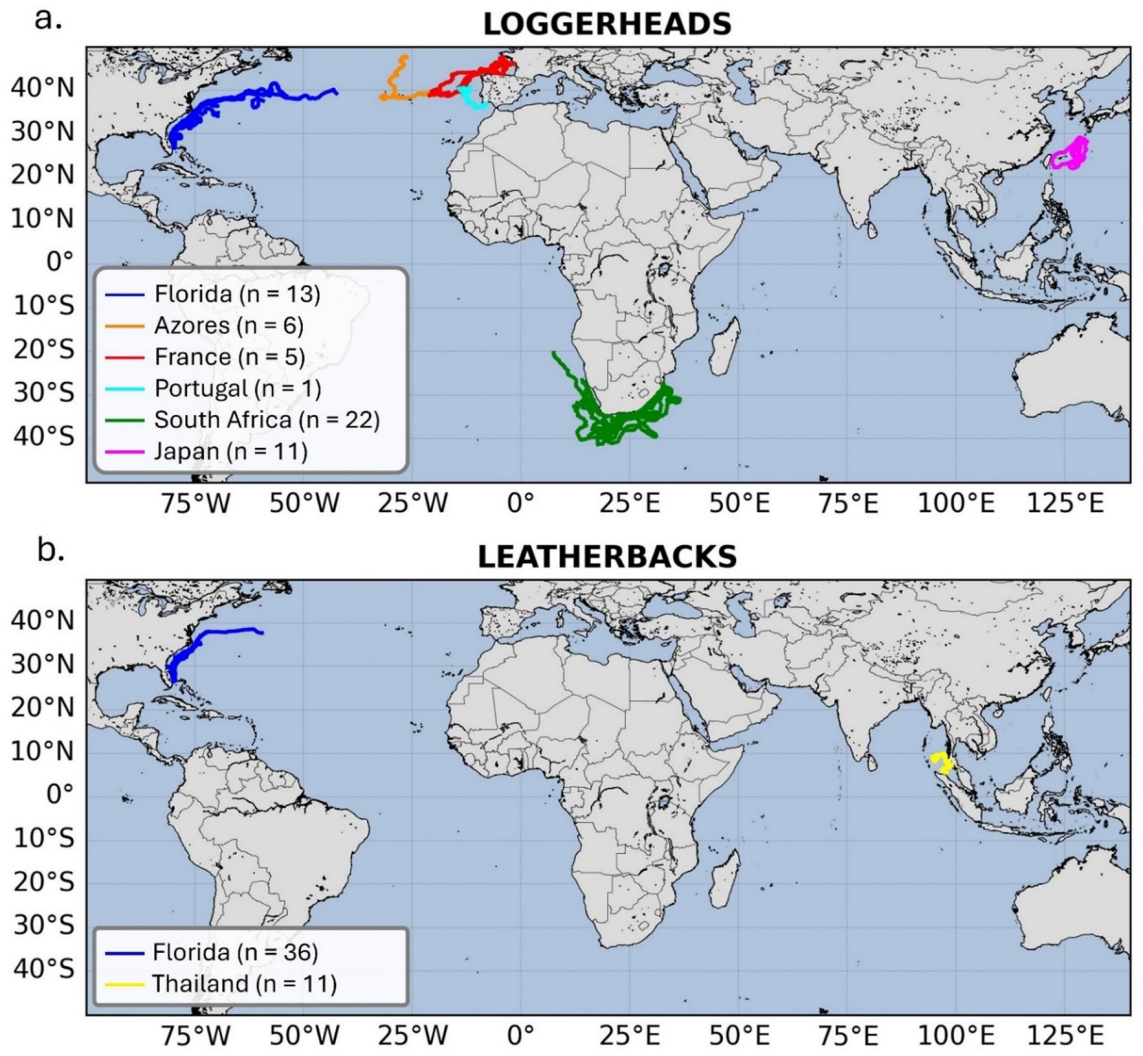
Trajectories of released sea turtles. Maps representing all the trajectories of loggerhead (**a**) and leatherback (**b**) sea turtles released in this study. Trajectories are color-coded depending on the release site with Eastern Florida (blue), the Azores archipelago (orange), the Bay of Biscay (red), Southern Portugal (cyan), South Africa (green), Southern Japan islands (magenta) and Western Thailand (yellow).

As of February 26th, 2025, tracking data were downloaded for all individuals involved in this study (Fig. 2) and are presented here as mean ± SD. The sea turtles’ tracking duration averaged 32.2 ± 40.9 days. Five tags deployed on loggerheads were still transmitting at this time, four of them for more than 100 days and one for more than 200 days. Tracking durations were significantly longer for loggerheads (50.8 ± 46.1 days) than for leatherbacks (9.3 ± 12.9 days). The reasons for this difference in tracking durations, likely reflecting the combination of demanding species-specific behaviors and the lack of robustness of the tag and its attachment, are discussed by Candela et al. (2024).

Strict quality control of the dive data compelled us to discard the complete dive records of 34 individuals (see details in supplementary materials S2, S3). The final edited dataset includes dive records from 71 sea turtles (42 loggerheads and 29 leatherbacks) for a total of about 2,500 daily values of each analyzed variable (Table 1). Less than 10% of the daily values were from leatherbacks because they were fewer within our dataset and had shorter tracking durations than the loggerheads. In addition, tags deployed on leatherbacks exhibited a lower transmission ratio (46%) than those on loggerhead sea turtles (76%), as previously noticed and discussed by Candela et al.^12^.

**Table 1:** Composition of the final dataset.

Species	Size interval (cm)	Origin	Number of turtles	Number of TaD distributions	Number of %TU	Number of M3D	Number of complete daily dive summaries
Loggerhead	Small (9.0 < SCL < 15.0)	Captive-reared	21	748	813	813	748
		Rehabilitated Wild-caught	13	832	848	846	830
		Total	34	1580	1661	1659	1578
	Intermediate (15.0 ≤ SCL < 21.0)	Captive-reared	1	72	74	74	72
		Rehabilitated Wild-caught	5	500	503	500	497
		Total	6	572	577	574	569
	Large (26.0 ≤ SCL ≤ 28.0)	Rehabilitated Wild-caught	2	97	102	100	95
Leatherback	Small (7.0 < SCL < 13.0)	Captive-reared	27	175	217	217	175
	Large (21.0 < SCL < 33.0)	Captive-reared	2	23	25	24	22
Total			71	2447	2582	2574	2439

Each individual is classified depending on species, origin (i.e., wild-caught/rehabilitated, or captive-reared), and size categories (SCL) at the time of release. For each category, the number of daily dive data is reported for the Time-at-Depth (TaD) distributions, the fractions of time spent underwater (%TU), the Maximum Daily Diving Depth (M3D) and the complete daily dive summaries (i.e., days for which all the aforementioned diagnoses were received).

### Vertical behavior characterization for loggerhead sea turtles

**Fig. 3:**
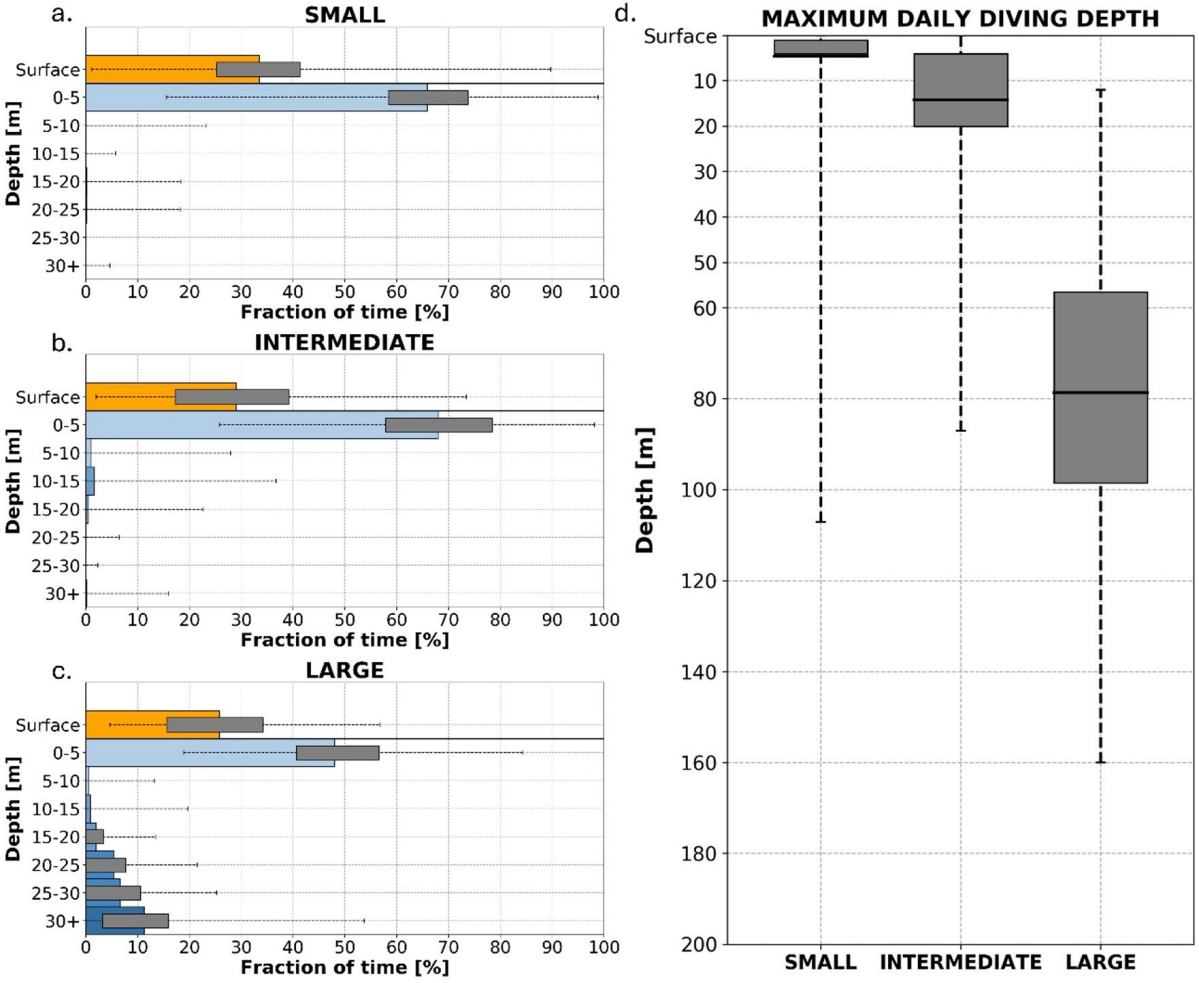
Vertical diving behavior of loggerhead sea turtles. On the left (**a**–**c**): mean Time-at-Depth distributions for small (**a**), intermediate (**b**) and large (**c**) loggerhead size classes. Each colored horizontal bar represents the mean fraction of time spent in the different vertical layers with the surface (orange) and 5-m depth bins (shades of blue). The lowest (dark blue) horizontal bar corresponds to the time spent at all depths below 30 m. On each, the interquartile range is represented by grey horizontal bars, as well as the total range of daily values (dashed line). On the right (**d**): Maximum Daily Diving Depth (M3D) distribution for each size class. The horizontal solid line represents the average M3D, the vertical grey bar shows the interquartile range, and the total range is represented by the vertical dashed line.

The smallest loggerheads (9.0 < SCL < 15.0 cm) exhibited strongly surface-oriented behavior with a mean %DT of only 0.3% (CI95 0.2–0.4%) and over 80% of M3Ds within the top 5 m of the water column (mean M3D ± SD: 4.5 ± 9.1 m) (Fig. 3a and d). Observed use of the water column confirmed this trend with individuals spending less than 0.5% of the day at depths greater than 5 m (Fig. 3a). Occasional deep dives were recorded, with 1% of M3Ds exceeding 50 m and a maximum dive recorded down to 107 m (Fig. 3d). This strongly surface-oriented behavior was similar in both captive-reared and rehabilitated individuals, although the latter showed slightly higher averages (mean %DT [CI95]: 0.6% [0.4–0.8%]; mean M3D ± SD: 5.4 ± 12.0 m) than the former (mean %DT [CI95]: 0.0% [0.0-0.1%]; mean M3D ± SD: 3.6 ± 4.3 m). However, this difference was driven by a single outlier (a rehabilitated loggerhead), which was released off South Africa where it dove longer and deeper than the other turtles, on average (mean %DT [CI95]: 10.3% [7.3–13.3%]; mean M3D ± SD: 29.8 ± 14.8 m). When excluding this individual outlier, average behavior converged (mean %DT [CI95]: 0.1% [0.0–0.2%]; mean M3D ± SD: 4.3 ± 10.6 m). Yet, the variability remained greater among rehabilitated turtles than among captively reared turtles, especially for M3D, highlighting inter-individual variability as a key differentiating factor.

Intermediate-sized loggerheads (15.0 ≤ SCL < 21.0 cm), released either from Florida (*n* = 1), France (*n* = 5), or South Africa (*n* = 2), also remained predominantly surface-oriented but showed a slight increase in dive depth and, in particular, a greater variability. Mean %DT increased to 3.1% (CI95 2.3–3.8%) with daily values ranging from 0.0 to 65.0% (Fig. 3b). Similarly, mean M3D rose from 4.5 m (SD: 9.1 m) to 14.2 m (SD: 14.0 m) with daily values ranging from 0.0 to 87.0 m and less than 30% of them occurring shallower than 5 m (Fig. 3d). Time in the upper layers remained dominant, but use of intermediate depths slightly increased to 0.9% (CI95 0.7–1.2%) between 5 and 10 m and 1.5% (CI95 1.1–1.9%) between 10 and 15 m (Fig. 3b). Time spent below 15 m remained below 0.5%. Interestingly, 40% of M3Ds were deeper than 15 m. This usage of the water column indicates that while deeper dives can occur, they were generally rare and brief.

The largest juvenile loggerheads (26.0 ≤ SCL ≤ 28.0 cm) exhibited a distinct shift in vertical behavior relative to the two other size classes. They spent a greater proportion of each day (measured as mean %DT [CI95]) diving (26.5% [23.2–29.8%]) (Fig. 3c), and diving more deeply: mean M3D increased to 78.6 m (SD: 31.8 m, range: 12.0–160.0 m). Over 80% of the M3D values were deeper than 50 m and 25% were deeper than 100 m (Fig. 3d). These turtles used the entire sampled vertical range, with all sampled depths spanning more than 0.5% of the day. They remained near the surface most of the time (mean %DT [CI95]: 26.5% [23.2–29.8%]), yet also, uniquely, exhibited a strong bimodal use of the water column. Intermediate depths were largely bypassed, with an average of 0.6% (CI95 0.2-1.0%) of the time spent between 5 and 10 m and 0.9% (CI95 0.3–1.4%) between 10 and 15 m, while use of depths below 15 m increased, with an average of 1.9% (CI95 1.3–2.5%) of the time spent between 15 and 20 m, 5.4% (CI95 4.3–6.5%) between 20 and 25 m, 6.5% (CI95 5.3–7.8%) between 25 and 30 m, and 11.2% (CI95 9.0-13.4%) beyond 30 m (Fig. 3c). 95% of M3D values from the largest loggerheads exceeded 30 m, supporting their bimodal usage of the water column.

### Vertical behavior characterization for leatherback sea turtles

**Fig. 4:**
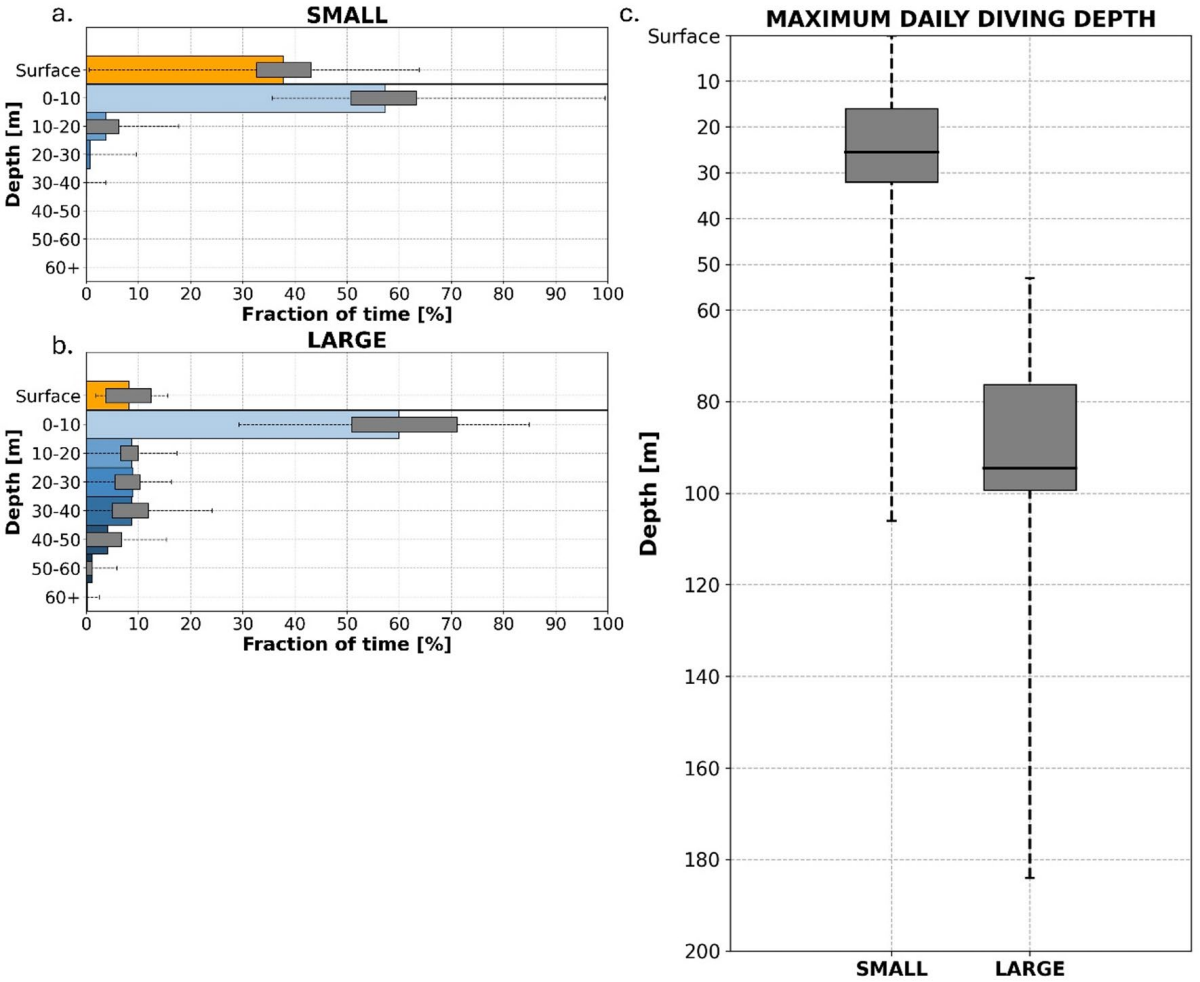
Vertical diving behavior of juvenile leatherback sea turtles. On the left: mean Time-at-Depth distributions for small (**a**) and large (**b**) leatherback size classes. Each colored horizontal bar represents the mean fraction of time spent in the different vertical layers with the surface (orange) and 10-m depth bins (shades of blue). The last (dark blue) horizontal bar corresponds to the time spent at all depths below 60 m. On each, the interquartile range is represented by grey horizontal bars, as well as the total range of daily values (dashed line). On the right: Maximum Daily Diving Depth (M3D) distribution (**c**) for each size class. The horizontal solid line represents the average M3D, the vertical grey bar shows the interquartile range, and the total range is represented by the vertical dashed line.

Small leatherbacks displayed a surface-oriented behavior with an average %DT of 4.5% (CI95 3.6–5.4%) and never exceeded 25% in a single day (Fig. 4a). However, they routinely dove beyond 10 m with 84% of days recording M3D deeper than 10 m and an average M3D of 25.4 m (SD: 15.7 m). The deepest recorded dive for this size class was 106 m (Fig. 4c). The use of the water column confirmed a surface habitat but also indicated that deeper dives were relatively frequent. The small juvenile leatherbacks spent on average 3.8% (CI95 3.0–4.5%) of their time between 10 and 20 m, 0.7% (CI95 0.4–1.0%) between 20 and 30 m and less than 0.5% in greater depths yet not exceeding 40 m in mean depth. The data support surface-oriented to relatively shallow depth habitat, punctuated by brief but frequent dives, mainly between 10 and 30 m (Fig. 4a).

Data from the largest leatherbacks showed a dramatically different profile from the small leatherbacks. The larger juveniles spent significantly more time diving, with a mean %DT rising from 4.5% (CI95 3.6–5.4%) in small leatherbacks to 31.5% (CI95 25.5–37.6%) in the large leatherbacks (Fig. 4b). Large juveniles also had much deeper dives compared to the small leatherbacks with an M3D average of 94.5 m (SD: 33.6 m, range: 53.0–184.0 m) (Fig. 4c). Large juvenile individuals still exhibited an important use of the near-surface layer of the water column (mean time spent in the first 10 m of the water column [CI95]: 59.9% [53.3–66.5%]), but on average, they spent less time directly at the surface: at 8.1% (CI95 6.1–10.1%). Instead, large juvenile leatherbacks spent more time at greater depths with an average of 8.7% (CI95 7.0–10.3%) of the time spent between 10 and 20 m, 8.9% (CI95 7.1–10.6%) between 20 and 30 m, 8.7% (CI95 6.2–11.1%) between 30 and 40 m, 4.1% (CI95 2.1–6.0%) between 40 and 50 m and 1.1% (CI95 0.2–2.0%) between 50 and 60 m (Fig. 4b). More than 95% of M3D values were deeper than 60 m, but time use below 60 m was less than 0.5%, suggesting that deep dives were relatively short but recurrent.

### Variation of the vertical behavior with size at the time of the release

The GAMMs revealed a significant (p-values < 0.001) relationship between body size (SCL) at the time of the release and vertical behavior for loggerheads and for leatherbacks (Table 2). In loggerheads, %DT and M3D remained low for the smallest individuals and increased with size, especially for turtles larger than 15 cm (Figure S4-1). In leatherbacks, similar results were found and, while %DT showed a pronounced increase for the larger individuals, M3D increased more continuously with size across the observed range, showing an almost linear relationship between the smallest and the largest turtles (Figure S4-1).

**Table 2:** Statistical analysis of vertical behavior in relation to body size at the time of the release.

Species	Response variables	Estimated degrees of freedom for turtle size	Reference degrees of freedom for turtle size	Statistics	*p*-value
Loggerhead	%DT	2.821	2.887	434.101	< 0.001
	M3D	2.952	2.961	34.520	< 0.001
Leatherback	%DT	2.312	2.482	26.470	< 0.001
	M3D	1.281	1.486	56.740	< 0.001

Results from Generalized Additive Mixed Models (GAMMs) assessing the effect of the Straight Carapace Length (SCL) at the time of the release on the fraction of time spent diving (%DT) and the Maximum Daily Diving Depth (M3D), analyzed separately for each species. For %DT, smooth terms were characterized using chi-squared statistics, whereas for M3D, smooth terms were characterized using F-statistics.

### Variation of the vertical behavior with time elapsed since the release

**Fig. 5:**
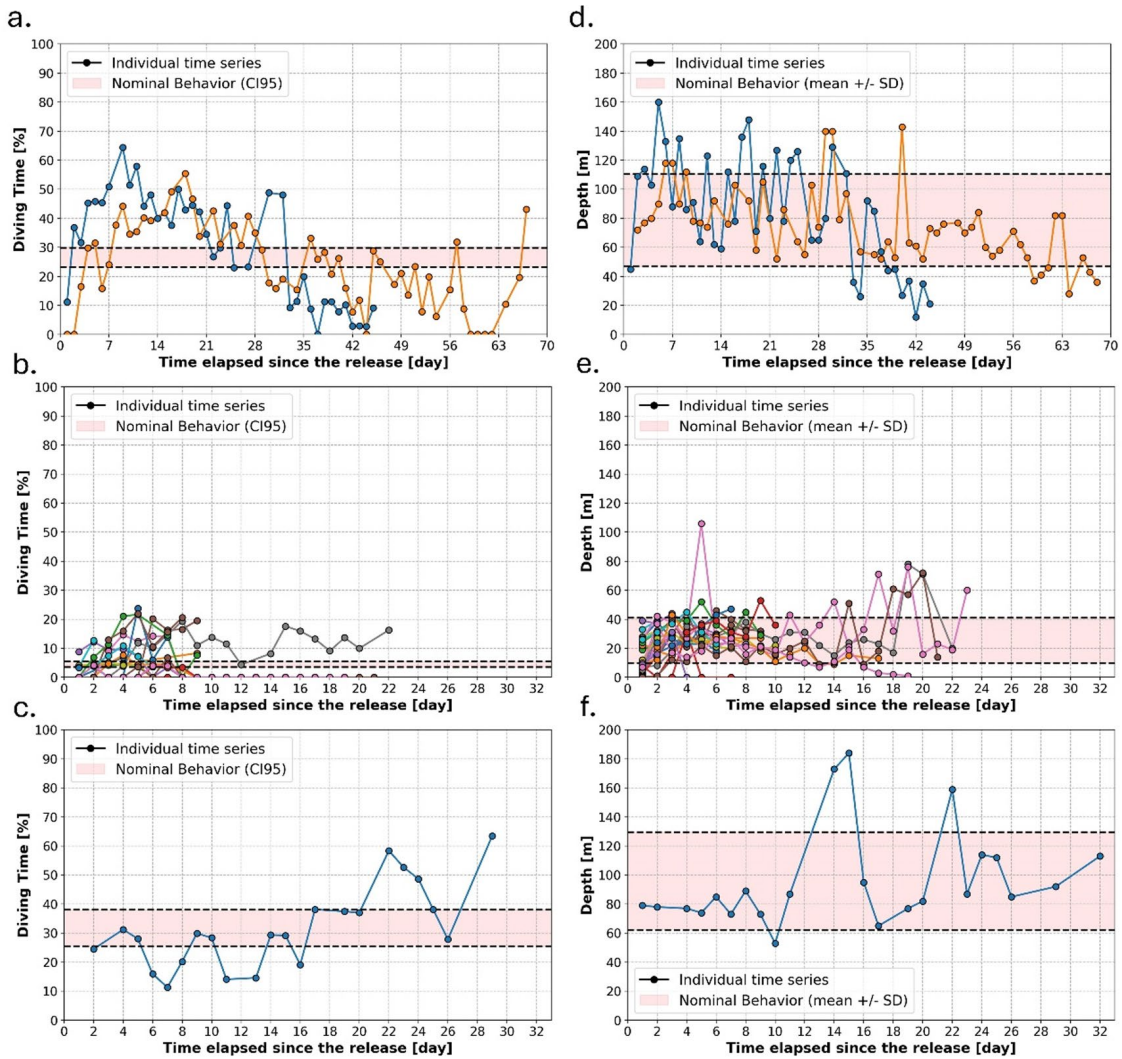
Individual time series of diving time (%DT) and Maximum Daily Diving Depth (M3D). **a**–**c** show individual time series of %DT for the two large rehabilitated loggerheads (**a**), small juvenile leatherbacks (**b**), and the single larger juvenile leatherbacks with multi-day data (**c**). Panels (d-f) show the corresponding M3D time series. On each panel, the nominal behavior range is represented (in red) as the 95% confidence interval (CI95) for %DT and as the average plus or minus one standard deviation for M3D. Nominal behavior thresholds are represented by black dashed lines. Individual time series: colored dots represent different individuals.

Small and intermediate-sized loggerheads showed consistently minimal vertical activity with very little variation throughout the tracking period, as supported by averages presented above. In contrast, the two largest loggerheads (rehabilitated animals) displayed a progressive increase in their %DT and M3D over the first weeks at sea, followed by a gradual decline (Fig. 5a and d).

For leatherbacks, during their first days at sea, most individuals exhibited low %DT and M3D (Fig. 5b and e). On Day 1, 95% of individuals had %DT values below the class average (4.5%), with low inter-individual variability (CI95 0.1–0.2%). Over Days 2–4, both the variability and the proportion of individuals exceeding the average steadily increased, as did their M3D values. By Day 3, 94% of turtles had reached the nominal M3D range, which remained stable for the following two weeks. Deeper dives beyond 60 m in small leatherbacks were mostly observed after this two-week period, though these were short or sporadic, and did not lead to increased %DT values. Interestingly, the only large leatherback with multi-day data did not exhibit an initial adjustment phase and instead exhibited nominal %DT and M3D values from the beginning (Fig. 5c and f). However, deeper dives beyond 100 m also emerged only after two weeks, and unlike the smaller turtles, these were accompanied by increased %DT, suggesting longer or more sustained activity.

The GAMMs similarly revealed contrasting relationships between time elapsed since release and vertical diving behavior in loggerhead and leatherback turtles (Table 3). While the %DT was not significantly related to time elapsed since the release for loggerheads (p-value: 0.571), a significant relationship was found between %DT and the time elapsed since the release for leatherbacks (p-value: 0.004). More precisely, leatherbacks exhibited very low values of %DT during approximately the first 4 days after their release, followed by a stabilization phase and a further increase after about 20 days at sea (Figure S5-1). The M3D was significantly related to the time elapsed since the release for both species when considering the full range of tracking durations (p-values: < 0.05). In that context, M3D from loggerheads remained relatively stable during the first 50 days at sea and then increased to a peak around 130 days, before a further decrease. Because this late increase, occurring nearly 2 months after the release, is unlikely to reflect post-release acclimation processes, the analysis was repeated using only the first 30 days of each loggerhead track. Within this restricted period, no significant relationship between M3D and the time elapsed since release was detected for loggerheads (p-value: 0.402). In contrast, leatherback turtles showed a significant (p-value: 0.04) and approximately linear increase in M3D over the duration of their tracks (i.e. up to about 30 days).

**Table 3:** Statistical analysis of vertical behavior in relation to the time elapsed since the release.

Species	Response variables	Estimated degrees of freedom for time elapsed since release	Reference degrees of freedom for time elapsed since release	Statistics	*p*-value
Loggerhead	%DT	1.005	1.010	0.329	0.571
	M3D	2.971	2.999	38.730	< 0.001
	M3D (< 30 days)	1.000	1.001	0.704	0.402
Leatherback	%DT	2.498	2.816	13.410	0.004
	M3D	1.000	1.000	8.480	0.004

Results from Generalized Additive Mixed Models (GAMMs) assessing the effect of the time elapsed since the release on the fraction of time spent diving (%DT) and the Maximum Daily Diving Depth (M3D), analyzed separately for each species. For %DT, smooth terms were characterized using chi-squared statistics, whereas for M3D, smooth terms were characterized using F-statistics.

### Variation of the vertical behavior with environmental variables

In contrast with the small and intermediate-sized juvenile loggerheads, which exhibited very limited vertical activity without any associations with environmental drivers such as temperature, SWH, or chl-a concentration, interesting observations were made for the two largest individuals of this species within our tracking dataset. Small and intermediate-sized loggerheads consistently maintained a surface-oriented behavior regardless of ocean conditions, but the two largest individuals within our dataset displayed two distinct phases of vertical behavior, which may correspond to contrasting ocean conditions.

**Fig. 6:**
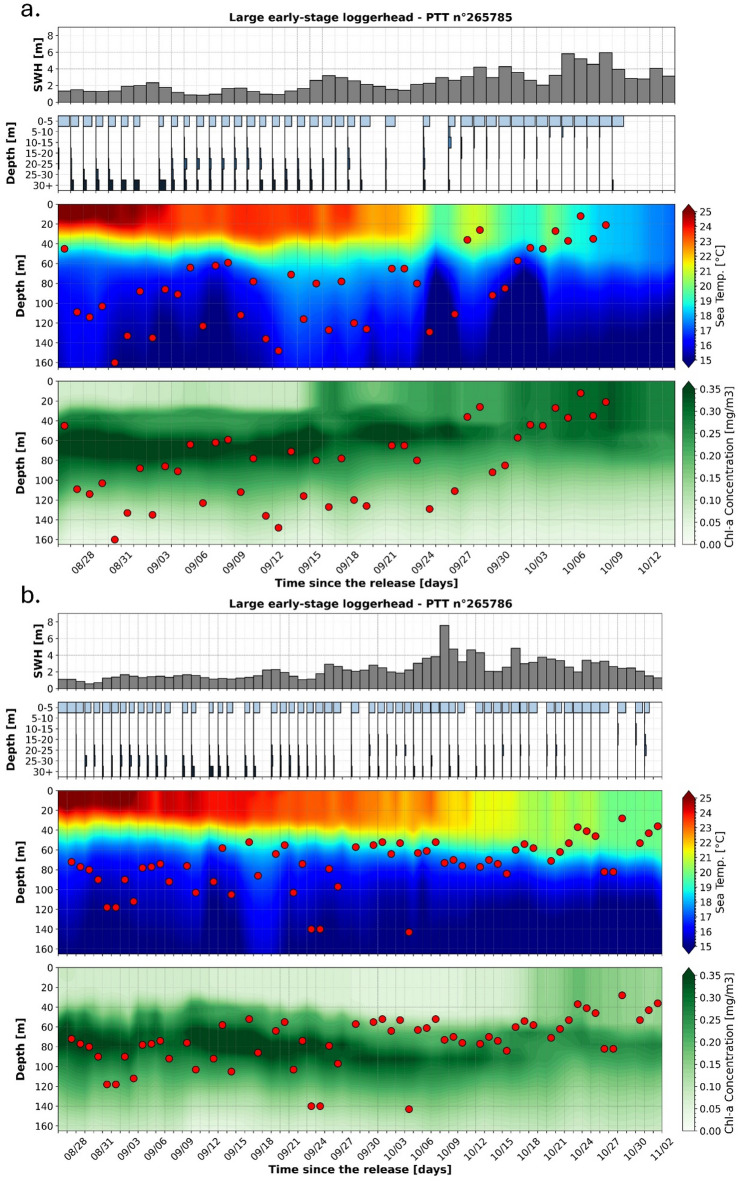
Significant wave height, Time-at-Depth (TaD) distributions and vertical profiles of sea water temperatures and chlorophyll-a concentrations along the two largest juvenile loggerhead trajectories. Significant wave heights (first/top panels), Time at Depth (TaD) distributions (second panels), vertical distributions of sea water temperature (third panels) and vertical distributions of chlorophyll-a concentrations (fourth/bottom panels) along the trajectories of the two largest loggerhead sea turtles equipped with tags n°265785 (**a**) and n°265786 (**b**). On third and fourth panels, Maximum Daily Diving Depth (M3D) are represented as red dots.

First, the two largest juvenile loggerhead turtles of this study were released in the Azores during late summer (on August 25th, 2024) during a very warm sea surface temperature (SST) episode for the area. During the first month of the tracking period, a warm surface mixed layer (SST between 21.2 and 26.1 °C) was clearly observed. Its depth was stable (close to 30 m) with a sharp thermocline beneath it, suggesting minimal vertical mixing between the mixed layer and the underlying thermocline. A well-marked chl-a maximum was present within the thermocline, typically between 40 and 60 m (Fig. 6). During this period, the two large loggerheads exhibited a clear bimodal use of the water column, being predominantly within the first 5 m of the water column (mean [CI95]: 63.4% [59.7–67.1%]) or below 20 m (mean [CI95]: 34.4% [30.8–38.0%]). These turtles also performed daily deep dives, reaching an average M3D of 95.0 m (SD: 28.0 m) (Fig. 6). The water temperature at these depths was close to 16.5 °C, the water temperature difference between the surface and the M3D being close to a 7.5 °C differential.

The upper ocean started to cool down with the arrival of autumn and SST progressively decreased below 20 °C. The temperature gradient between the upper mixed layer and the thermocline decreased markedly, facilitating turbulent vertical mixing and deepening of the mixed layer. This vertical mixing led to a more uniform distribution of chl-a in the water column as it brought the chl-a from the enriched thermocline waters into the mixed layer (Fig. 6). Within the same timeframe, the vertical movements of the two large juvenile loggerheads changed to a predominantly surface-oriented behavior (Fig. 6). They spent an average of 35.0% (CI95 31.5–38.6%) of their time at the surface and 50.3% (CI95 47.3–53.3%) within the first 5 m of 50.3% (CI95 47.3–53.3%). Lower depths were rarely but evenly used. On average, the two individuals spent only 1.2% (CI95 0.4–2.1%) of their time between 5 and 10 m, 1.5% (CI95 0.4–2.6%) between 10 and 15 m, 1.9% (CI95 1.0-2.9%) between 15 and 20 m, 3.8% (CI95 2.3–5.4%) between 20 and 25 m, 2.2% (CI95 1.4–3.1%) between 25 and 30 m, and 4.1% (CI95 2.8–5.4%) below 30 m. In addition to their surface-oriented behaviors, deep dives were also less frequent during that period and the average M3D decreased to 59.4 m (SD: 24.6 m). For comparison purposes, this seasonal shift in vertical behavior was not observed in small and intermediate-sized individuals. In both warm (SST > = 24 °C) and in colder (SST < 24 °C) waters, these turtles remained almost exclusively at or near the surface with a mean fraction of time spent above 5 m of more than 95% for both the smallest and the intermediate-sized individuals.

**Fig. 7:**
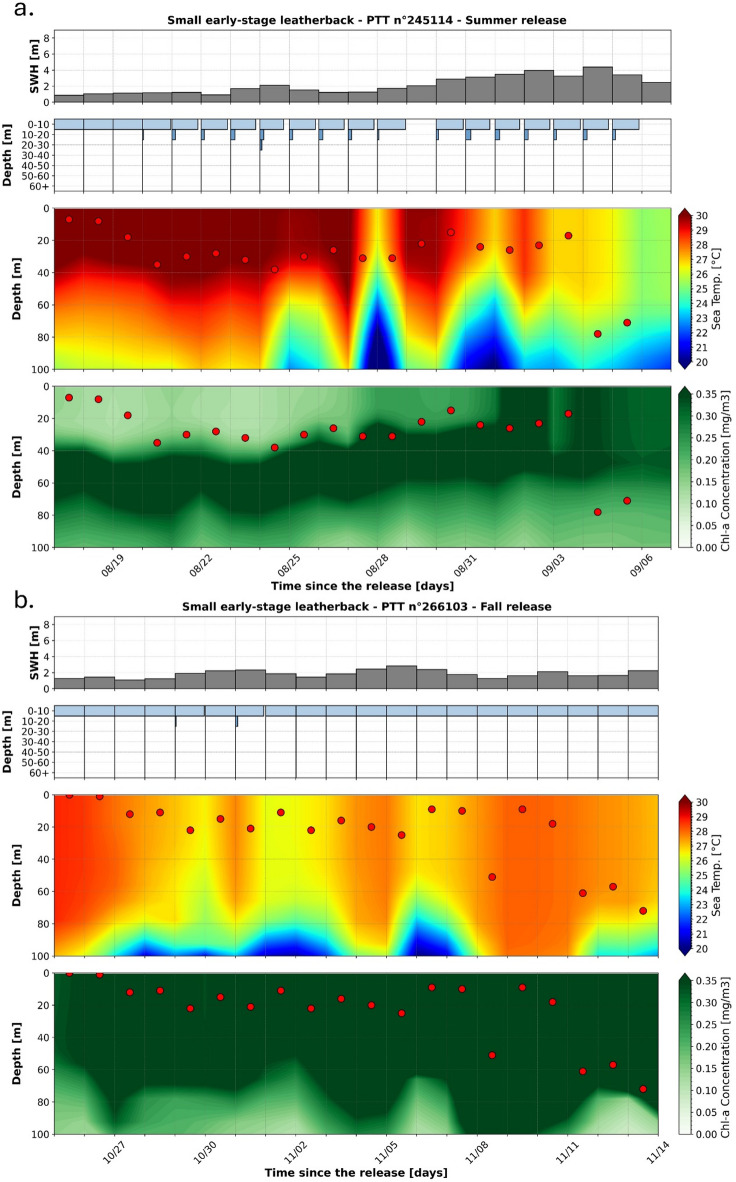
Significant wave height, Time at Depth distributions and vertical profiles of sea water temperatures and chlorophyll-a concentrations along the two longest small leatherback trajectories, released in the summer or in the autumn season. Significant wave heights (first/top panels), Time at Depth (TaD) distributions (second panels), vertical distributions of sea water temperature (third panels) and vertical distributions of chlorophyll-a concentrations (fourth/bottom panels) along the two longest trajectories of small leatherback sea turtles equipped with tags n°245,114 (a), which was released during the summer season, and n°266,103 (b), which was released during the autumn season. On third and fourth panels, Maximum Daily Diving Depth (M3D) are represented as red dots.

Small leatherbacks were all released during either the summer or the fall season. They dispersed within the very warm waters of the Gulf Stream’s environment and, consequently, they experienced an average SST of 28.9 °C (SD: 1.8 °C) along their trajectories. Despite these very warm surface conditions, they mostly remained near the surface, as reflected by their low average %DT of 4.5% (CI95 3.6–5.4%). Only a small seasonal difference was observed in their vertical behaviors with a higher fraction of time spent diving during summer months (August-September) than during autumn months (October-November) (Fig. 7). Indeed, the average %DT decreases from 7.3% (CI95 5.9–8.6%) during summer months to 0.8% (CI95 0.4–1.2%) during autumn months. These small juvenile leatherbacks jointly performed relatively deep dives on a daily basis as shown by their average M3D of 25.4 m (SD: 15.7 m) but no clear seasonal variation was observed between summer and autumn months with a mean M3D of 27.7 m (SD: 14.1 m) and 22.1 m (SD: 17.2 m), respectively (Fig. 7). Whatever the season, the surface waters were warm (average SST higher than 27.0 °C for both seasons). Because of the deep thermocline (60–100 m) of the Gulf Stream, the temperatures encountered while diving were barely different from the surface with average temperature differences smaller than 1.0 °C between the surface and these depths for both seasons (Fig. 7). Despite no clear seasonal difference in the maximum depths reached by the turtles, one interesting observation is that while the water column was stratified during summer months, with low surface chl-a concentration and a well‑defined deep maximum roughly between 30 and 70 m (Fig. 7a); during the autumn season, the chl-a concentration was relatively uniform and high throughout the whole turtles’ diving range (Fig. 7b). In that context, while the deep dives performed during the autumn season remained within the mixed and nutrient-rich surface layer, the deep dives performed in summer generally terminated just above the deep chl-a maximum (Fig. 7).

**Fig. 8:**
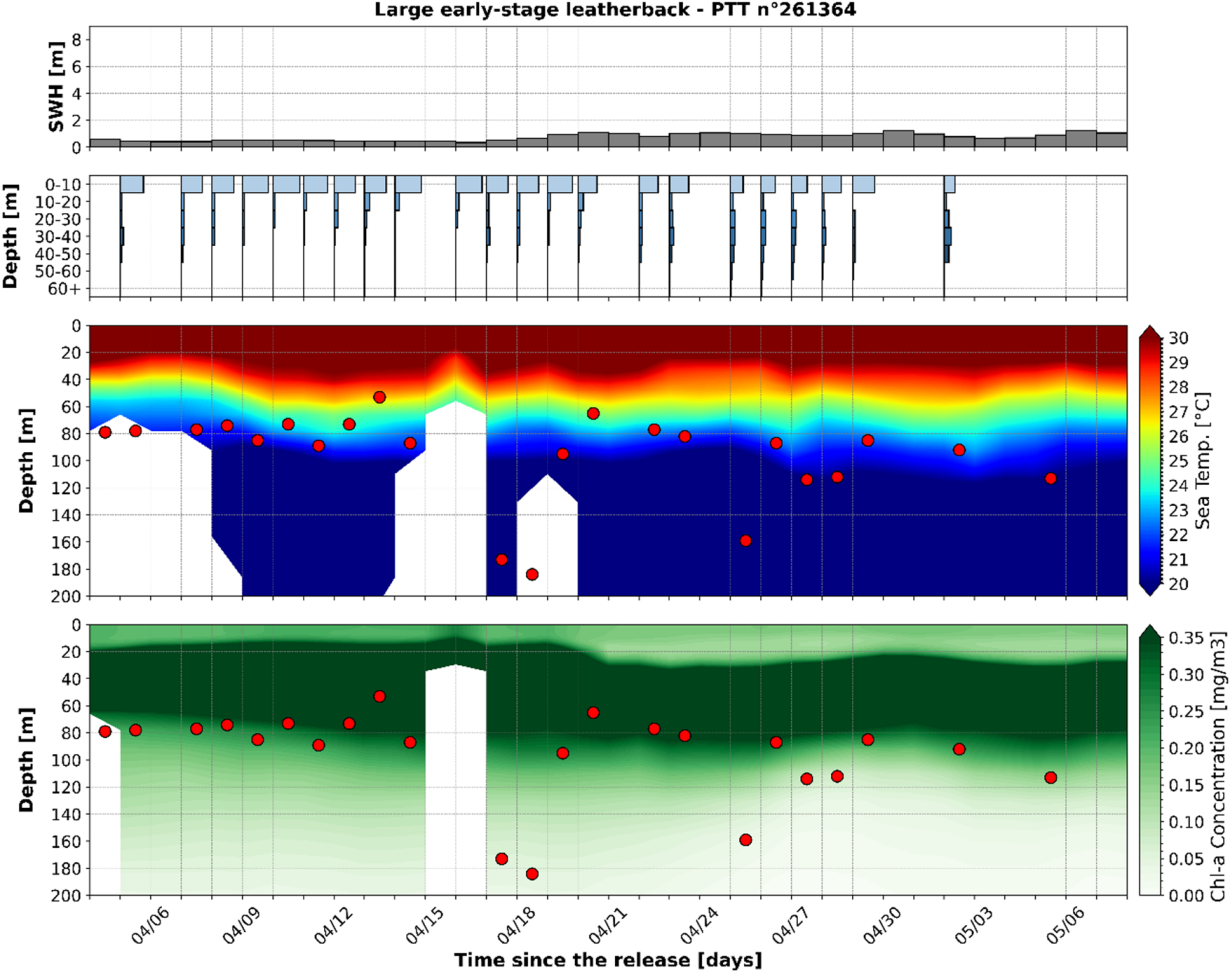
Significant wave height, Time at Depth distributions and vertical profiles of sea water temperatures and chlorophyll-a concentrations along the selected large early-stage leatherback trajectory. Significant wave heights (first/top panel), Time at Depth (TaD) distributions (second panel), vertical distributions of sea water temperature (third panel) and vertical distributions of chlorophyll-a concentrations (fourth/bottom panel) along the trajectory of the large early-stage leatherback sea turtle equipped with tags n°261,364. On third and fourth panels, Maximum Daily Diving Depth (M3D) are represented as red dots and blank spaces indicate absence of data where modeled bathymetry is shallower than 200 m.

In contrast with the smallest leatherbacks, the vertical activity of the only large early-stage leatherback (SCL: 27.60 cm) revealed that this individual experienced much colder water temperatures than at the surface. This turtle was released during early April (on April 2nd, 2024), within the very warm waters of the Andaman Sea, and experienced an average SST of 31.8 °C (SD: 0.4 °C) (Fig. 8). For the entire tracking period, the water column was thermally well stratified with a 20 to 30 m deep mixed layer with water temperatures over 30 °C. While the turtle spent over 85% of its time above 10–20 m, M3Ds, generally in the range of 50 to 120 m were observed every day, and occasionally reached depths below 150 m. At these M3Ds, the mean water temperature encountered at M3Ds was close to 21 °C and the average temperature difference between the surface and the M3D was 10.6 °C, much larger than the temperature differences experienced by the small leatherbacks. These deep dives systematically reached or exceeded the lower bound of the deep chl-a maximum, which seems to seasonally plunge by mostly starting around 10–15 m during the first 18 days of the tracking period and around 20–25 m afterwards. In the same timeframes, the fraction of time spent in the upper 20 m of the water column decreases from 86.7% (CI95 82.3–91.1%) during the first 18 days of the tracking period, to 62.9% (CI95 53.4–72.3%) afterwards.

## Discussion

### Ontogenetic shifts in vertical behavior

These findings demonstrate clear ontogenetic shifts in the extent and complexity of vertical habitat use and diving behavior for both juvenile loggerhead and leatherback sea turtles. Our results showed that as individuals age and grow (i.e., as SCL at the time of the release increase), their diving abilities appear to increase, with larger turtles spending more time diving, reaching greater depths, and showing a greater proportion of sustained sub-surface activity. These ontogenetic changes in the vertical behavior of young sea turtles are consistent with the development of their physiological capacities, notably the increase in oxygen storage and buoyancy control^38–43^. Newly hatched sea turtles initially lack fine control over their specific buoyancy due to immature musculature and relatively small lung volume. Consequently, they are physiologically constrained to remaining at the surface^39^. However, as they grow, buoyancy control improves with the development of necessary physiological adaptations, such as lung capacity and musculature, which impact oxygen storage, aerobic diving capacity and ventilation efficiency. These physiological changes enable older juveniles to sustain longer and deeper dives with lower energetic costs^38,40,41,43–46^. Thus, the ontogenetic changes in dive behavior observed in this study could be explained by physiological changes that occur as the turtles develop and grow.

As size increased, the use of the water column became more extensive and structured. While the smaller individuals remained primarily in the upper part of the water column and exhibited surface-dominant behavior, larger individuals adopted a broader vertical strategy that involved consistent use of greater ocean depths. Large juvenile loggerheads routinely exploited depths beyond 30 m and showed bimodal use of the water column, concentrated near the surface and in greater depths with minimal use of intermediate depths. This bimodal vertical pattern, already observed in much larger oceanic juveniles (SCL: 35–65 cm) in the North Atlantic and Pacific Ocean^47,48^, suggests a targeted use of the water column, with intermediate layers likely serving as a transit zone between the surface, where individuals mostly breathe and rest, and a preferred deeper layer. As previously reported for larger juvenile and adult loggerhead sea turtles, preferences may be induced by higher prey density, thermoregulatory needs, or optimization of swimming conditions^47–52^. In leatherbacks, we observed similar ontogenetic shifts, with the larger individuals diving more frequently and to greater depths with much less time spent at the surface than the smallest individuals. As diving capacity increases with growth, leatherback sea turtles are able to use the water column more extensively and flexibly^53,54^. Consequently, the need to rest at the surface after each dive likely diminishes and while surfacing remains essential for breathing, the reduced surface residency in larger turtles may indicate a decreased reliance on surface recovery periods, enabling longer submersion periods. This phenomenon has been observed in other marine vertebrates^55^, although the underlying physiological mechanisms likely differ. Leatherbacks should gain access to a wider range of depths as they grow, and therefore, to a wider range of foraging resources. Adult leatherback sea turtles are known to forage throughout the water column, exploiting gelatinous zooplankton that occur from surface waters to well below the epipelagic zone^56–61^. The increasing vertical range observed in larger juvenile leatherbacks in this study aligns with this ecological role, suggesting that as physiological constraints relax with growth, individuals begin to access a greater diversity of foraging opportunities throughout the water column.

### Acclimation to a new environment

After spending months in shallow and controlled environments during captive rearing or rehabilitation, some juvenile sea turtles may require a short post-release acclimation period. This transitional phase likely allows individuals to (re)discover their diving capacities, adjust to the open ocean, and recover from potential stress related to captivity, handling, or tagging^62^.

Overall, the data suggests the existence of a short post-release acclimation period of about 4 days, especially for the smallest leatherback turtles. Once acclimated, individuals settled into a consistent diving routine for a week or two and, in some cases, began exploring greater ocean depths afterward. This pattern was not observed in the loggerheads of this dataset.

### Environmental drivers of vertical behavior

Environmental conditions such as sea state, temperature gradients, prey distribution and predation risk are known to influence the vertical behavior of marine megafauna, including sea turtles^63–71^. In particular, thermoregulatory and foraging strategies have been proposed as key drivers shaping diving patterns across life stages and species^50,61,72^.

In our dataset, small and intermediate-sized juvenile loggerheads remained largely confined to or near the surface, with deep dives occurring only rarely and showing very limited variations across ocean conditions. This conforms to the generally surface-oriented behavior described in the literature for oceanic juvenile loggerheads^39,47,48,50^, while still reflecting physiological limits that restrict the extent of their vertical movements^39^. In contrast, noteworthy observations have been made with the other individuals, including the two largest loggerheads and the leatherbacks. However, given the limited number of individuals and the complexity of the multi-dimensional relationships between vertical behavior and the three-dimensional structure of the environment over time, the associations described below are presented as descriptive observations only. They cannot be interpreted as demonstrated environmental drivers but may help generate hypotheses for future studies.

The two largest loggerheads exhibited seasonally variable vertical behavior. During the first month following their release in summer, both turtles showed a bimodal use of the water column, within highly stratified waters. This vertical behavior enabled them to experience strong thermal gradients and to access waters much cooler than at the surface, close to their presumed preferred temperature around 18 °C^73,74^. As surface waters cooled later in the season, the water column mixed and this vertical behavior shifted toward a mostly surface-oriented distribution. A similar seasonal signal was observed in small leatherbacks, spending a greater fraction of time diving during summer than during autumn. However, for these individuals, the deep dives did not expose them to large thermal gradients. The only large juvenile leatherback for which multiple days of dive data were received only experienced warm water conditions. Although it remained predominantly in the upper and very warm 30 m, this turtle frequently dived to about 100 m, experiencing strong thermal gradients and consistently reaching waters between 22 and 24 °C, which could be close to its preferred body temperature^75^. Such vertical patterns observed in warm surface waters and seasonal changes may evoke thermoregulatory processes. However, given the small sizes (SCL < 30 cm) and low body masses (< 5 kg) of these early-stage juvenile loggerheads and leatherbacks, their limited thermal inertia likely constrains the extent to which sustained thermoregulation through diving can be achieved^65–67^. Sea water temperature alone may not account for the observed vertical behavior.

In addition, observations involving vertical distribution of chl-a, used here as a proxy for prey availability, may point toward a potential link with foraging strategies. Deep chl-a maximums are known to concentrate biodiversity (predators and prey alike) much like a deep scattering layer^76^. Dives reaching or extending beyond this layer may thus reflect active targeting of these prey-rich habitats, perhaps after initial exploratory dives and subsequent strategies^61,69,76^. As autumn progresses and vertical mixing disrupts the stratification that likely supported these aggregations^77–79^, chl-a and prey tend to become more evenly distributed throughout the water column and a more surface-oriented vertical behavior was observed.

Altogether, these observations indicate that the vertical behavior of early-stage juvenile sea turtles may reflect interactions with multiple aspects of their physical and biological environment. While thermal structure and prey distribution may both be relevant to the observed patterns, the present dataset does not allow their respective influences to be disentangled or quantified. Concurrent oceanographic measurements and prey sampling in these habitats would help to elucidate the finer scale drivers of juvenile sea turtle vertical behavior.

This study provides the most extensive and detailed description to date of vertical behaviors in very early-stage juvenile loggerhead and leatherback sea turtles, revealing key ontogenetic and behavioral patterns that shape how these individuals interact with their three-dimensional environment. By improving our understanding of mechanisms during the Lost Years, this work offers a rare empirical foundation for anticipating juvenile movements in the open ocean and for assessing the conditions under which young turtles may encounter anthropogenic threats. These insights can support the development of more realistic three-dimensional representations of juvenile behavior in dispersal frameworks and can ultimately inform conservation strategies and guide the formulation of three-dimensional marine protected areas, reducing incidental mortality risks and optimizing conservation strategies for the most vulnerable stages of the sea turtle life cycle.

## Supplementary Information

Below is the link to the electronic supplementary material.

Supplementary Material 1.

## Data Availability

Restrictions apply to the availability of these data. Data were obtained from Upwell Turtles and are part of an ongoing collaborative research program involving multiple institutions. Due to ongoing analyses and planned publications, the full dataset is not publicly available at this stage. However, data are available upon reasonable request to the corresponding author and George L. Shillinger (george@upwell.org ), with permission from Upwell Turtles.
