# Supplementary material for: Pioneering insights into the diving behavior of early-stage sea turtles revealed by novel marine miniaturized satellite tags
Source: Sci Rep. 2026 Apr 9;16:16692. doi: 10.1038/s41598-026-47239-6 (PMC13219612; doi:10.1038/s41598-026-47239-6)
Supplement: Supplementary file 1 — Supplementary Material 1. [file 41598_2026_47239_MOESM1_ESM.docx]

Pioneering insights into the diving behavior of early-stage sea turtles revealed by novel marine miniaturized satellite tags

Tony Candela^1,2,3*^, Philippe Gaspar^2^, Helen Bailey^4^, Jeanette Wyneken^5^, Emily Turla^5^, Talitha Noble-Trull^6^, Ronel Nel^7^, Hirun Kanghae^8^, Pinsak Suraswadi^9^, Tipamat Upanoi^10^, Junichi Okuyama^11^, Isao Kawazu^12,13^, Ken Maeda^13^, Kaho Mizuochi^13^, Nene Ogino^13^, Frederic Vandeperre^14,15^, Ana Mafalda Sousa^14,15^, Andrea Herguedas^14,15^, Mark De Boer^16^, Florence Dell’Amico^3^, Antonieta Nunes^17^, Joao Neves^17^, Isabel Gaspar^17^, and George L. Shillinger^1^

^1^Upwell, Monterey, California, United States of America

^2^Mercator Ocean International, Toulouse, France

^3^Aquarium La Rochelle, Centre d’Etudes et de Soins pour les Tortues Marines, La Rochelle, France

^4^Blue Wave Consulting, LLC, Baltimore, Maryland, United State of America

^5^FAU Marine Science Laboratory, Department of Biological Sciences, Florida Atlantic University, Boca Raton, Florida, United States of America

^6^Two Oceans Aquarium Foundation, Cape Town, South Africa

^7^Nelson Mandela University, Port Elizabeth, South Africa

^8^Phuket Marine Biological Center, Department of Marine and Coastal Resources, Phuket, Thailand

^9^Department of Marine and Coastal Resources, Bangkok, Thailand

^10^Marine and Coastal Resources Research Center (Upper Andaman Sea), Phuket, Thailand

^11^Sea Turtle Ecology Lab, Yokohama, Kanagawa, Japan

^12^Okinawa Churashima Foundation, Okinawa, Japan

^13^Okinawa Churaumi Aquarium, Okinawa, Japan

^14^Institute of Marine Sciences, IICM Okeanos, University of the Azores, 9901-862 Horta, Portugal

^15^Institute of Marine Research, IMAR, 9900-138 Horta, Portugal

^16^Rotterdam Zoo, Rotterdam, The Netherlands

^17^Zoomarine Algarve, Mundo Aquatico SA, Guia, Albufeira, Portugal

^*^Corresponding author

Email: tcandela@mercator-ocean.fr (Tony Candela)

Keywords: juvenile sea turtles, vertical behavior, diving activity, miniaturized satellite tags, foraging strategy, thermoregulation, migration

**S1: Sea turtle details**

| ID | Species | Release SCL [cm] | Tag model | Release area | Release date | Origin | Tracking duration [day] |
| --- | --- | --- | --- | --- | --- | --- | --- |
| 263645 | Caretta caretta | 14.40 | K4H 130B Dive | Azores | 2024 Jul. 17 | Wild-caught | 1 |
| 263646 | Caretta caretta | 13.50 | K4H 130B Dive | Azores | 2024 Jul. 17 | Wild-caught | 1 |
| 263647 | Caretta caretta | 13.40 | K4H 130B Dive | Azores | 2024 Jul. 17 | Wild-caught | < 1 |
| 263648 | Caretta caretta | 11.20 | K4H 130B Dive | Azores | 2024 Jul. 17 | Wild-caught | 52 |
| 265785 | Caretta caretta | 28.00 | K4G 132A Sensor | Azores | 2024 Aug. 25 | Rehabilitated | 50 |
| 265786 | Caretta caretta | 26.00 | K4G 132A Sensor | Azores | 2024 Aug. 25 | Rehabilitated | 70 |
| 236302 | Dermochelys coriacea | 7.47 | K4H 130B Dive | Florida | 2022 Aug. 10 | Captive-reared | 10 |
| 236305 | Dermochelys coriacea | 8.90 | K4H 130B Dive | Florida | 2022 Aug. 10 | Captive-reared | < 1 |
| 236310 | Dermochelys coriacea | 7.42 | K4H 130B Dive | Florida | 2022 Aug. 10 | Captive-reared | 8 |
| 236303 | Dermochelys coriacea | 9.47 | K4H 130B Dive | Florida | 2022 Sep. 22 | Captive-reared | 10 |
| 236306 | Caretta caretta | 9.44 | K4H 130B Dive | Florida | 2022 Sep. 22 | Captive-reared | 4 |
| 236308 | Caretta caretta | 10.08 | K4H 130B Dive | Florida | 2022 Sep. 22 | Captive-reared | 15 |
| 236309 | Caretta caretta | 9.14 | K4H 130B Dive | Florida | 2022 Sep. 22 | Captive-reared | 31 |
| 245110 | Dermochelys coriacea | 8.59 | K4H 130B Dive | Florida | 2023 Aug. 16 | Captive-reared | 7 |
| 245111 | Dermochelys coriacea | 8.91 | K4H 130B Dive | Florida | 2023 Aug. 16 | Captive-reared | 9 |
| 245112 | Dermochelys coriacea | 9.39 | K4H 130B Dive | Florida | 2023 Aug. 16 | Captive-reared | 10 |
| 245113 | Dermochelys coriacea | 8.95 | K4H 130B Dive | Florida | 2023 Aug. 16 | Captive-reared | 8 |
| 245114 | Dermochelys coriacea | 8.42 | K4H 130B Dive | Florida | 2023 Aug. 16 | Captive-reared | 22 |
| 245115 | Dermochelys coriacea | 9.11 | K4H 130B Dive | Florida | 2023 Aug. 16 | Captive-reared | 6 |
| 245116 | Dermochelys coriacea | 9.42 | K4H 130B Dive | Florida | 2023 Aug. 16 | Captive-reared | 9 |
| 245117 | Dermochelys coriacea | 8.43 | K4H 130B Dive | Florida | 2023 Aug. 16 | Captive-reared | 3 |
| 245118 | Dermochelys coriacea | 8.65 | K4H 130B Dive | Florida | 2023 Aug. 16 | Captive-reared | 3 |
| 245119 | Dermochelys coriacea | 8.22 | K4H 130B Dive | Florida | 2023 Aug. 16 | Captive-reared | < 1 |
| 245120 | Dermochelys coriacea | 9.11 | K4H 130B Dive | Florida | 2023 Aug. 16 | Captive-reared | 1 |
| 245121 | Dermochelys coriacea | 9.36 | K4H 130B Dive | Florida | 2023 Aug. 16 | Captive-reared | 9 |
| 245122 | Dermochelys coriacea | 9.40 | K4H 130B Dive | Florida | 2023 Sep. 20 | Captive-reared | < 1 |
| 245124 | Dermochelys coriacea | 9.34 | K4H 130B Dive | Florida | 2023 Sep. 20 | Captive-reared | 2 |
| 245125 | Dermochelys coriacea | 9.72 | K4H 130B Dive | Florida | 2023 Sep. 20 | Captive-reared | < 1 |
| 245126 | Dermochelys coriacea | 8.94 | K4H 130B Dive | Florida | 2023 Sep. 20 | Captive-reared | 2 |
| 245127 | Dermochelys coriacea | 8.71 | K4H 130B Dive | Florida | 2023 Sep. 20 | Captive-reared | 8 |
| 245128 | Caretta caretta | 10.60 | K4H 130B Dive | Florida | 2023 Sep. 20 | Captive-reared | 13 |
| 245129 | Caretta caretta | 10.58 | K4H 130B Dive | Florida | 2023 Sep. 20 | Captive-reared | 4 |
| 245130 | Dermochelys coriacea | 9.45 | K4H 130B Dive | Florida | 2023 Sep. 20 | Captive-reared | 24 |
| 245131 | Dermochelys coriacea | 9.00 | K4H 130B Dive | Florida | 2023 Sep. 20 | Captive-reared | 3 |
| 245132 | Dermochelys coriacea | 9.23 | K4H 130B Dive | Florida | 2023 Sep. 20 | Captive-reared | 11 |
| 245133 | Dermochelys coriacea | 9.54 | K4H 130B Dive | Florida | 2023 Sep. 20 | Captive-reared | 7 |
| 266092 | Dermochelys coriacea | 9.13 | K4H 130B Dive | Florida | 2024 Oct. 24 | Captive-reared | 6 |
| 266093 | Dermochelys coriacea | 9.55 | K4H 130B Dive | Florida | 2024 Oct. 24 | Captive-reared | 17 |
| 266094 | Dermochelys coriacea | 9.13 | K4H 130B Dive | Florida | 2024 Oct. 24 | Captive-reared | 2 |
| 266097 | Dermochelys coriacea | 11.09 | K4H 130B Dive | Florida | 2024 Oct. 24 | Captive-reared | 10 |
| 266098 | Dermochelys coriacea | 9.60 | K4H 130B Dive | Florida | 2024 Oct. 24 | Captive-reared | < 1 |
| 266099 | Dermochelys coriacea | 9.14 | K4H 130B Dive | Florida | 2024 Oct. 24 | Captive-reared | < 1 |
| 266100 | Dermochelys coriacea | 10.80 | K4H 130B Dive | Florida | 2024 Oct. 24 | Captive-reared | 1 |
| 266101 | Dermochelys coriacea | 9.45 | K4H 130B Dive | Florida | 2024 Oct. 24 | Captive-reared | < 1 |
| 266102 | Dermochelys coriacea | 9.00 | K4H 130B Dive | Florida | 2024 Oct. 24 | Captive-reared | 4 |
| 266103 | Dermochelys coriacea | 12.10 | K4H 130B Dive | Florida | 2024 Oct. 24 | Captive-reared | 21 |
| 266105 | Dermochelys coriacea | 9.05 | K4H 130B Dive | Florida | 2024 Oct. 24 | Captive-reared | 19 |
| 264090 | Caretta caretta | 12.31 | K4H 130B Dive | Florida | 2024 Nov. 26 | Captive-reared | 11 |
| 264091 | Caretta caretta | 12.54 | K4H 130B Dive | Florida | 2024 Nov. 26 | Captive-reared | 41 |
| 264092 | Caretta caretta | 12.52 | K4H 130B Dive | Florida | 2024 Nov. 26 | Captive-reared | 72 |
| 264093 | Caretta caretta | 13.24 | K4H 130B Dive | Florida | 2024 Nov. 26 | Captive-reared | 39 |
| 264094 | Caretta caretta | 12.79 | K4H 130B Dive | Florida | 2024 Nov. 26 | Captive-reared | 43 |
| 264095 | Caretta caretta | 12.54 | K4H 130B Dive | Florida | 2024 Nov. 26 | Captive-reared | < 1 |
| 264098 | Caretta caretta | 12.10 | K4H 130B Dive | Florida | 2024 Nov. 26 | Captive-reared | 38 |
| 264099 | Caretta caretta | 16.20 | K4H 130B Dive | Florida | 2024 Nov. 26 | Captive-reared | 76 |
| 263650 | Caretta caretta | 20.40 | K4G 132A Sensor | France | 2024 Jul. 24 | Rehabilitated | 133 |
| 263651 | Caretta caretta | 18.20 | K4G 132A Sensor | France | 2024 Jul. 24 | Rehabilitated | 116 |
| 263652 | Caretta caretta | 16.90 | K4G 132A Sensor | France | 2024 Jul. 24 | Rehabilitated | 216* |
| 263653 | Caretta caretta | 16.40 | K4G 132A Sensor | France | 2024 Jul. 24 | Rehabilitated | 72 |
| 263654 | Caretta caretta | 17.50 | K4G 132A Sensor | France | 2024 Jul. 24 | Rehabilitated | 36 |
| 269179 | Caretta caretta | 11.54 | K4G 132A Sensor | Japan | 2024 Nov. 14 | Captive-reared | 87 |
| 269180 | Caretta caretta | 10.90 | K4G 132A Sensor | Japan | 2024 Nov. 14 | Captive-reared | 42 |
| 269181 | Caretta caretta | 11.26 | K4G 132A Sensor | Japan | 2024 Nov. 14 | Captive-reared | 98 |
| 269182 | Caretta caretta | 11.40 | K4G 132A Sensor | Japan | 2024 Nov. 14 | Captive-reared | 19 |
| 269183 | Caretta caretta | 11.40 | K4G 132A Sensor | Japan | 2024 Nov. 14 | Captive-reared | 24 |
| 269184 | Caretta caretta | 11.16 | K4G 132A Sensor | Japan | 2024 Nov. 14 | Captive-reared | 92 |
| 269185 | Caretta caretta | 11.60 | K4G 132A Sensor | Japan | 2024 Nov. 14 | Captive-reared | 36 |
| 269186 | Caretta caretta | 11.10 | K4G 132A Sensor | Japan | 2024 Nov. 14 | Captive-reared | 33 |
| 269188 | Caretta caretta | 12.10 | K4G 132A Sensor | Japan | 2024 Nov. 14 | Captive-reared | 102* |
| 269189 | Caretta caretta | 11.45 | K4G 132A Sensor | Japan | 2024 Nov. 14 | Captive-reared | 102* |
| 269190 | Caretta caretta | 11.40 | K4G 132A Sensor | Japan | 2024 Nov. 14 | Captive-reared | 44 |
| 264648 | Caretta caretta | 26.60 | K4G 132A Sensor | Portugal | 2024 Jul.26 | Rehabilitated | 146 |
| 253956 | Caretta caretta | 13.00 | K4G 132A Sensor | South Africa | 2024 Jan. 20 | Rehabilitated | 88 |
| 253957 | Caretta caretta | 15.00 | K4G 132A Sensor | South Africa | 2024 Jan. 20 | Rehabilitated | 29 |
| 253958 | Caretta caretta | 13.90 | K4G 132A Sensor | South Africa | 2024 Jan. 20 | Rehabilitated | 2 |
| 253959 | Caretta caretta | 13.90 | K4G 132A Sensor | South Africa | 2024 Jan. 20 | Rehabilitated | < 1 |
| 253960 | Caretta caretta | 16.20 | K4G 132A Sensor | South Africa | 2024 Jan. 20 | Rehabilitated | < 1 |
| 253961 | Caretta caretta | 11.90 | K4G 132A Sensor | South Africa | 2024 Jan. 20 | Rehabilitated | < 1 |
| 253962 | Caretta caretta | 13.70 | K4G 132A Sensor | South Africa | 2024 Jan. 20 | Rehabilitated | 4 |
| 253963 | Caretta caretta | 14.20 | K4G 132A Sensor | South Africa | 2024 Jan. 20 | Rehabilitated | 27 |
| 253964 | Caretta caretta | 13.40 | K4G 132A Sensor | South Africa | 2024 Jan. 20 | Rehabilitated | < 1 |
| 253965 | Caretta caretta | 13.30 | K4G 132A Sensor | South Africa | 2024 Jan. 20 | Rehabilitated | 11 |
| 266106 | Caretta caretta | 13.00 | K4G 132A Sensor | South Africa | 2024 Oct. 21 | Rehabilitated | 88 |
| 266107 | Caretta caretta | 12.70 | K4G 132A Sensor | South Africa | 2024 Oct. 21 | Rehabilitated | 97 |
| 266108 | Caretta caretta | 13.40 | K4G 132A Sensor | South Africa | 2024 Oct. 21 | Rehabilitated | 60 |
| 266109 | Caretta caretta | 14.90 | K4G 132A Sensor | South Africa | 2024 Oct. 21 | Rehabilitated | 127* |
| 266110 | Caretta caretta | 12.70 | K4G 132A Sensor | South Africa | 2024 Oct. 21 | Rehabilitated | 127* |
| 266111 | Caretta caretta | 13.50 | K4G 132A Sensor | South Africa | 2024 Oct. 21 | Rehabilitated | 32 |
| 266112 | Caretta caretta | 14.90 | K4G 132A Sensor | South Africa | 2024 Oct. 21 | Rehabilitated | 73 |
| 266113 | Caretta caretta | 14.40 | K4G 132A Sensor | South Africa | 2024 Oct. 21 | Rehabilitated | 104 |
| 266114 | Caretta caretta | 13.50 | K4G 132A Sensor | South Africa | 2024 Oct. 21 | Rehabilitated | 35 |
| 266115 | Caretta caretta | 13.30 | K4G 132A Sensor | South Africa | 2024 Oct. 21 | Rehabilitated | 43 |
| 266116 | Caretta caretta | 13.70 | K4G 132A Sensor | South Africa | 2024 Oct. 21 | Rehabilitated | 37 |
| 266117 | Caretta caretta | 13.70 | K4G 132A Sensor | South Africa | 2024 Oct. 21 | Rehabilitated | < 1 |
| 254016 | Dermochelys coriacea | 21.96 | K4G 132A Sensor | Thailand | 2024 Apr. 02 | Captive-reared | < 1 |
| 261359 | Dermochelys coriacea | 25.39 | K4G 132A Sensor | Thailand | 2024 Apr. 02 | Captive-reared | 64 |
| 261360 | Dermochelys coriacea | 32.50 | K4G 132A Sensor | Thailand | 2024 Apr. 02 | Captive-reared | < 1 |
| 261363 | Dermochelys coriacea | 28.21 | K4G 132A Sensor | Thailand | 2024 Apr. 02 | Captive-reared | 1 |
| 261364 | Dermochelys coriacea | 27.60 | K4G 132A Sensor | Thailand | 2024 Apr. 02 | Captive-reared | 36 |
| 261365 | Dermochelys coriacea | 27.81 | K4G 132A Sensor | Thailand | 2024 Apr. 02 | Captive-reared | 1 |
| 261366 | Dermochelys coriacea | 36.00 | K4G 132A Sensor | Thailand | 2024 Apr. 02 | Captive-reared | < 1 |
| 261367 | Dermochelys coriacea | 31.40 | K4G 132A Sensor | Thailand | 2024 Apr. 02 | Captive-reared | < 1 |
| 261368 | Dermochelys coriacea | 26.27 | K4G 132A Sensor | Thailand | 2024 Apr. 02 | Captive-reared | 40 |
| 261369 | Dermochelys coriacea | 25.74 | K4G 132A Sensor | Thailand | 2024 Apr. 02 | Captive-reared | 39 |
| 261370 | Dermochelys coriacea | 26.43 | K4G 132A Sensor | Thailand | 2024 Apr. 02 | Captive-reared | < 1 |

**Table S1-1: Detailed information for all individuals included in the study.**

Table summarizing detail for all individuals included in the study, including PTT ID, species, straight carapace length at the time of the release (in cm), tag model, release location and date, origin (i.e., captive-reared, rehabilitated, or wild-caught) and tracking duration (in days). Tracking durations with asterisk indicate PTT that were still transmitting at the time of the study.

**S2: Dive-related data filtering process**

After the standardization, dive-related data was filtered to homogenize the dataset and avoid erroneous data. First, data from the release day was removed from the dataset. Since the release occurred during the day, this daily summary was not based on a 24-hour period, from 00:00 to 23:59 UTC, and cannot be consistent with the other tracking days.

In order to avoid transmission errors, the first filtering process is the Parity Bit Control (PBC). Each transmission takes the form of a series of 7 bits containing information to be transmitted to which an eighth is added to detect a potential error. Here, the PBC is based on an even parity bit calculation, stating that the transmission needs to include an even number of “1” bits to be assessed as not corrupted.

The PBC is widely used as the first simple error-checking process, but it is not sufficient on its own, as the inversion of two bits causes the transmission to be considered as correct when it is not. In this regard, another controlling process, based on the recurrence of sequences of variables (taking into account Argos message compression), is applied on the dataset, the Majority Rule Control (MRC). The basic rule of this second process states that, for a given day (as the data is given through daily summary), the most frequent sequence of variables is the correct one and all the others are transmission errors. Overall, this process follows a series of 5 simple rules:

1. If only one sequence of variables was received and it has been received multiple times, the sequence is assessed as correct;
2. If only one sequence of variables was received and it has been received only once, the sequence is assessed as undefined;
3. If multiple sequences of variables were received and one of them was received more frequently than the others, the most recurrent sequence is assessed as correct while the others are assessed as corrupted;
4. If multiple sequences of variables were received and all of them have the same number of occurrences, they are all assessed as undefined;
5. If multiple sequences of variables were received and multiple sequences were received more frequently than the others, but these have the same number of occurrences, these more frequent sequences are assessed as undefined while the others are assessed as corrupted.

After classifying all the transmissions, only those assessed as correct were kept and all the others were removed from the dataset.

Incorrect information, because of measurement issues or equipment failures, can be correctly transmitted and thus, still in the dataset after the PBC and the MRC. This data was also filtered in order to avoid any bias in the final analysis. To do so, an outlier identification was applied on the data, aiming to highlight values that stand out from the others. Moreover, an additional method to identify erroneous M3D was applied on the dataset. M3D values were compared with the corresponding bathymetric value for that day. If the maximum daily diving depth exceeded the local bathymetry, the measurement was likely erroneous. However, because bathymetric data were extracted at the resampled daily locations (12:00 UTC), they may not precisely reflect the actual locations where the deepest dives occurred, which could take place up to 12 hours before or after the reference time. To account for this spatial uncertainty, a potential dispersal area was defined around each daily location. This area was represented by a circle with a radius equal to half the mean daily travel distance, calculated across the entire trajectory. Within this framework, the M3D was assumed to potentially occur anywhere within this dispersal area. Consequently, when a M3D value appeared to exceed the corresponding bathymetric depth, all bathymetric values within the dispersal area were examined. If at least one point within this area was deeper than the recorded M3D, the measurement was considered as correct. Otherwise, it was classified as erroneous.

Finally, the last filtering process was applied on the TaD distributions. Indeed, TaD distributions – relayed by Type 5 transmissions – only makes sense if the %TU over the 24-hour period – relayed Type 6 transmission as well as the M3D – was also received. Therefore, when TaD distributions were received without any associated %TU, the TaD was removed from the dataset.

**S3: Detailed results of the filtering process**

**Resampling of the trajectories**

Overall, after resampling the trajectories with aniMotum and excluding those lasting for 1 day or less (n = 25), a total of 3,360 tracking days were obtained from 80 sea turtles. Of these tracking days, 2,934 (87%) came from tags deployed on loggerhead sea turtles (n = 49), while 426 (13%) came from tags deployed on leatherback sea turtles (n = 31). This large difference in species representation is partly due to the much shorter tracking durations of leatherback sea turtles.

**Parity Bit Control**

From these tracking days, a total of 48,535 transmissions were received with almost the same proportion of T5 (n = 23,928 - 49%) and T6 (n = 24,607 – 51%). However, and as expected, most of them came from tags deployed on loggerhead sea turtles, with 43,304 transmissions (89%) against only 5,321 (11%) coming from tags deployed on leatherback sea turtles (Table S3-1).

|  | Number of transmissions | Number of corrupted transmissions | Number of correct transmissions |
| --- | --- | --- | --- |
| T5 | 23,928 | 1,113 | 22,815 |
| T6 | 24,607 | 759 | 23,848 |
| Loggerhead | 43,304 | 1,579 | 41,725 |
| Leatherback | 5,321 | 293 | 4,938 |
| ***Total*** | ***48,535*** | ***1,872*** | ***46,663*** |

**Table S3-1: Results of the Parity Bit Control.**

The Parity Bit Control (PBC) identified 1,872 corrupted transmissions, representing only 4% of the total number of transmissions. This corruption ratio is similar among transmission types with 5% for T5 and 3% for T6, and among equipped species with 4% for loggerhead and 6% for leatherback sea turtles. After the PBC, a total of 46,663 transmissions were still included in the dataset (Table S3-1).

**Majority Rule Control**

From these transmissions, a total of 6,327 sequences of variables were decoded with similar numbers of transmissions per sequence among transmission types and equipped species (Table S3-2). However, and as expected, most of the sequences of variables came from tags deployed on loggerhead sea turtles, with 5,677 sequences (90%) against only 650 (10%) coming from tags deployed on leatherback sea turtles.

|  | Number of transmissions | Number of sequences of variables | Number of transmissions per sequence |
| --- | --- | --- | --- |
| T5 | 22,815 | 3,169 | 7.20 |
| T6 | 23,848 | 3,158 | 7.55 |
| Loggerhead | 41,725 | 5,677 | 7.35 |
| Leatherback | 4,938 | 650 | 7.60 |
| ***Total*** | ***46,663*** | ***6,327*** | ***7.38*** |

**Table S3-2: Number of transmissions to decode sequences of variables.**

Out of these 6,327 sequences of variables, the Majority Rule Control (MRC) identified 769 (12%) as corrupted, 316 (5%) as undefined and 5,242 (83%) as correct (Table S3-3). While similar ratios were found among transmission types – with 13% of sequences assessed as corrupted, 5% as undefined and 83% as correct for T5, and with 11% of sequences assessed as corrupted, 5% as undefined and 83% as correct for T6 – this is not the case among species (Table S3-3). Indeed, while the fraction of sequences assessed as corrupted is similar for both species, with 12% for loggerhead sea turtles and 13% for leatherback sea turtles, tags deployed on leatherback sea turtles suffer from a more important number of sequences assessed as undefined than those deployed on loggerhead sea turtles with 10% and 4%, respectively. As a consequence, the fraction of sequences assessed as correct is higher for loggerhead sea turtles (84%) than for leatherback sea turtles (77%). Without the ability to determine the reliability of sequences assessed as undefined by the MRC, they, as well as those assessed as corrupted, were removed from the dataset. As a result of the MRC, the filtered dataset includes a total of 5,242 sequences of variables, almost evenly distributed between sequences coming from T5 (50%) and T6 (50%). However, the fraction of sequences coming from tags deployed on loggerhead sea turtles (90%) is still much more important than the sequences relayed by tags deployed on leatherback sea turtles (10%) (Table S3-3).

|  | Number of sequences of variables | Number of corrupted sequences | Number of undefined sequences | Number of correct sequences |
| --- | --- | --- | --- | --- |
| T5 | 3,169 | 408 | 146 | 2,615 |
| T6 | 3,158 | 361 | 170 | 2,627 |
| Loggerhead | 5,677 | 686 | 249 | 4,742 |
| Leatherback | 650 | 83 | 67 | 500 |
| ***Total*** | ***6,327*** | ***769*** | ***316*** | ***5,242*** |

**Table S3-3: Results of the Majority Rule Controls.**

**Programming errors**

Programming errors were detected on 6 tags, rendering the relayed data unsuitable for analysis. First, on one tag, the integration period for daily summary computing was different and was set from 12:00 UTC to 12:00 UTC the day after, rather than from 00:00 UTC to 00:00 UTC the day after. Consequently, data from this tag has been removed from the dataset. Second, on 5 tags, the depth bins parameterization was inverted between the loggerhead (5 meters) and the leatherback (10 meters) configurations. Therefore, as 2 tags were equipped on loggerhead sea turtles with the leatherback programming configuration and 3 tags were equipped on leatherback sea turtles with the loggerhead programming configuration, the data does not allow for consistent analysis with other individuals of their respective species. Consequently, only the TaD distributions transmitted from these tags have been removed from the dataset.

**Measurement errors and TaD dependency**

Focusing the filtering on T5, which relayed the TaD distributions, outliers were difficult to identify because of a high variability (Figure S3-4). Consequently, only 1 TaD distribution – transmitted by a tag deployed on loggerhead sea turtle and exhibiting constant values on 5 **
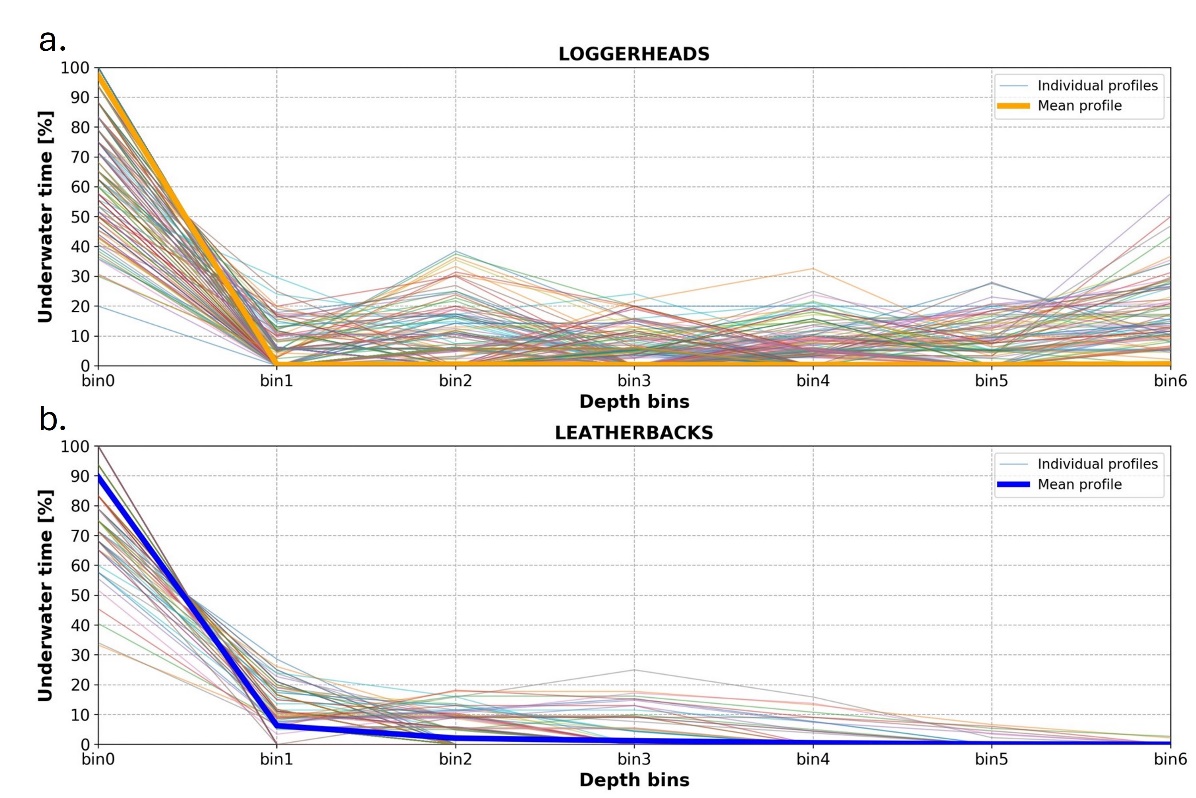
**out of the 7 depth bins – was identified as erroneous.

**Figure S3-4: Daily Time at Depth distributions for loggerhead and leatherback sea turtles.**

Fraction of underwater time spent in the different sampled depth bins for loggerhead (a) and leatherback sea turtles (b). Both, daily Time at Depth distributions (shaded lines) and the average Time at Depth distribution (bold line), were plotted for both species.

Then, focusing the analysis on T6, which relayed both M3D and %TU, multiple outliers were identified (Figure S2-2). For loggerhead sea turtles, %TUs were relatively well distributed between 10 and 100% and no outlier can be identified from it. However, multiple M3D were recorded over 400 meters and obviously appeared as outliers in the dataset (Figure S2-2a). For leatherback sea turtles, %TUs, and M3D were relatively well distributed between 35 and 100% and between 0 and 200 meters respectively. However, multiple transmissions indicated %TUs and M3D to 0% and 0 meters respectively (Figure S2-2b). All these transmissions were relayed by the same tag and therefore, it seems very likely that the sensor was defective and that these measurements are erroneous. In the identified outliers transmitted by loggerhead sea turtles, only the M3D was identified as erroneous and since it depends on a different sensor than the %TU, only this variable should be considered as well. Therefore, for these transmissions, only the M3D were removed from the dataset, but associated values of %TU were kept. For the outliers transmitted by the leatherback sea turtles, both variables were
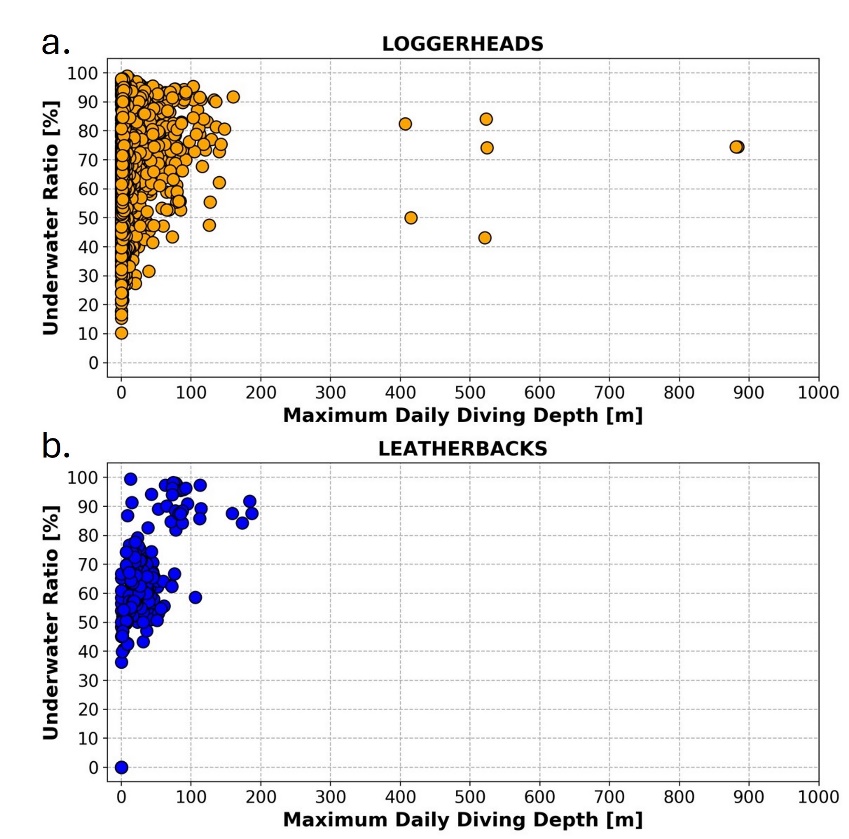
considered as erroneous and therefore, they were both removed from the dataset.

**Figure S3-5: M3D and %TU values for loggerhead and leatherback sea turtles.**

Daily values of %TU as a function of M3D values for loggerhead (a) and leatherback sea turtles (b).

The additional M3D measurement error checking process, based on local bathymetry values, identified a total of 8 M3D values exceeding the associated bathymetric value. However, after comparing the surrounding bathymetric values, only 1 was assessed as erroneous and was removed from the dataset. All the others had a consistent bathymetric value nearby.

On 74 tracking days, TaD distributions were correctly received without the associated %TU. Consequently, these TaD distributions are not suitable for analysis and were removed from the dataset.

**Dead turtle**

One loggerhead sea turtle released within the Azores ended its trajectory by 4 consecutive days with a M3D of 0 meters and 100% of the time spent near the surface before the transmission ceased. This specific behavior suggests that this turtle was floating for an undetermined reason (e.g., cold-stunning, injury after boat strike or predator encounter) before the tag stopped transmitting. As it could bias the results, these 4 days of data were also removed from the dataset.

**S4: Relationships between body size and vertical behavior based on generalized additive mixed model**

**
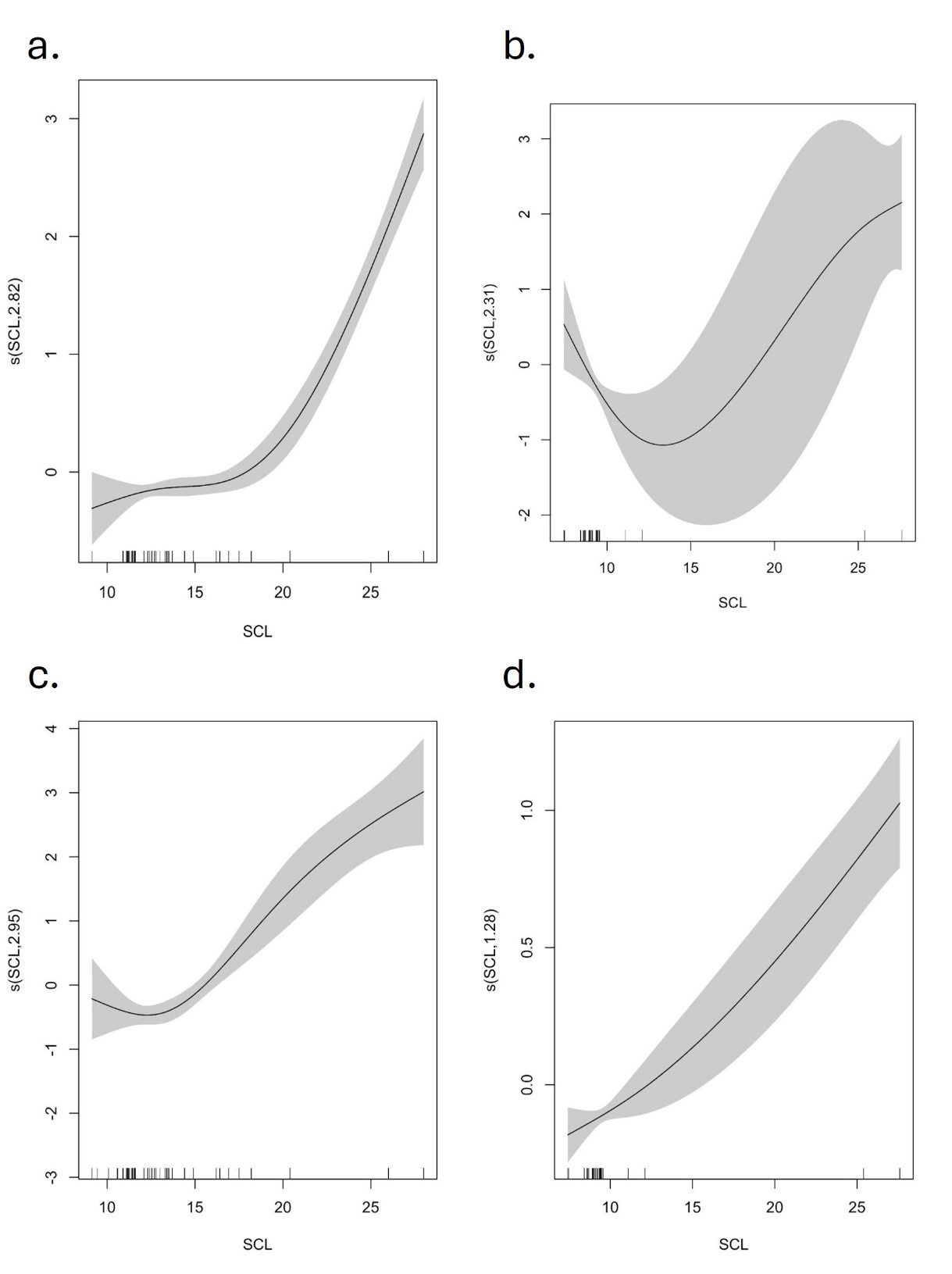
Figure S4-1: Size-dependent variations in vertical behavior.**

Generalized additive mixed model (GAMM) smoothers of the relationship between the vertical behavior and the straight carapace length (SCL) at the time of the release for loggerheads on the left column (a, c) and leatherbacks on the right column (b, d). Relationships with the fraction of time spent diving (%DT) are on the top line (a, b) and relationships with Maximum Daily Diving Depth (M3D) are on the bottom line (c, d). Confidence intervals are shown as grey shading and the black lines on the x-axis show the distribution of the observation data.

**S5: Relationships between time elapsed since the release and vertical behavior based on generalized additive mixed model**

**
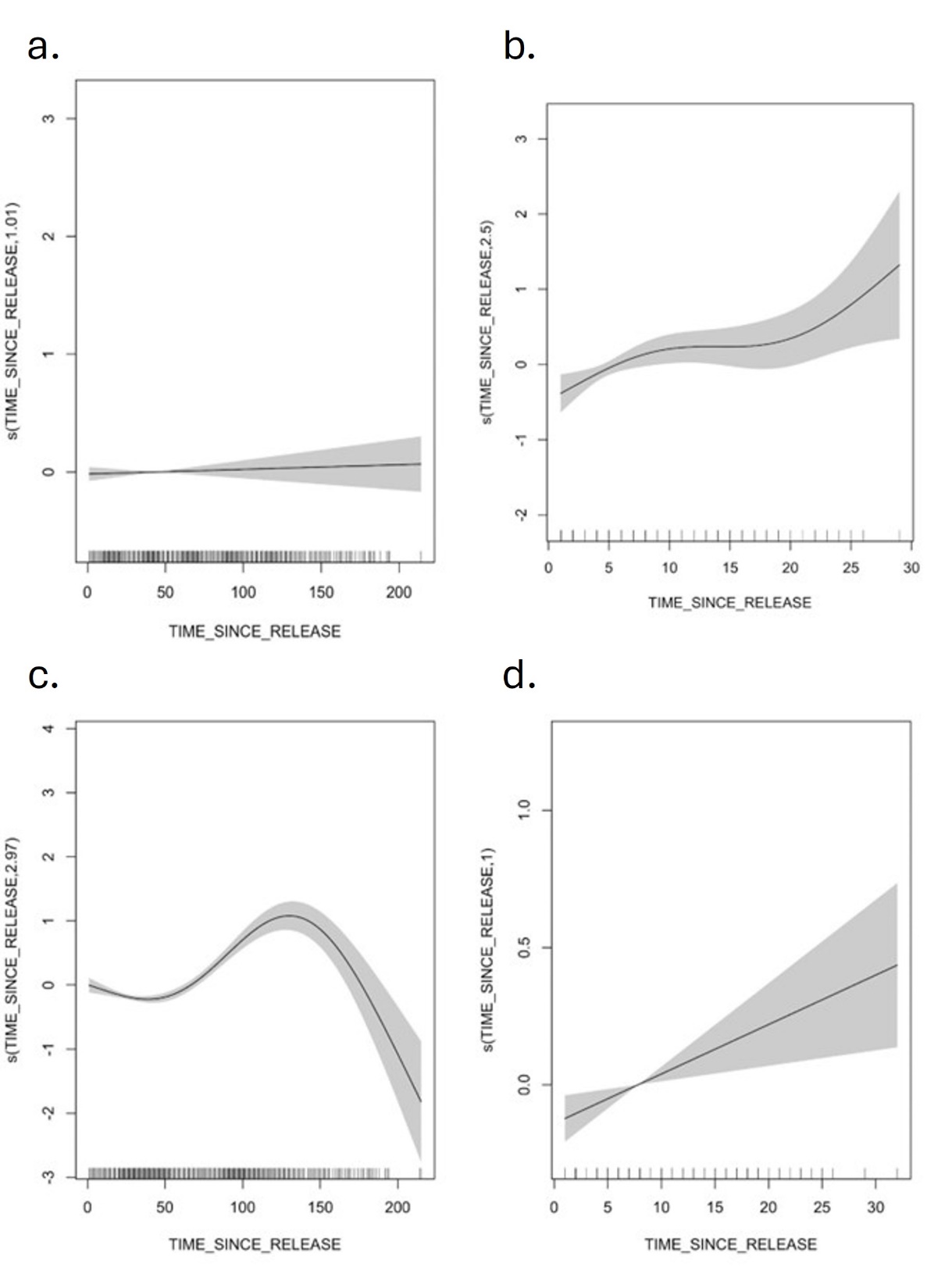
Figure S5-1: Variations in vertical behavior in relation to time elapsed since the release.**

Generalized additive mixed model (GAMM) smoothers of the relationship between the vertical behavior and the time elapsed since the release for loggerheads on the left column (a, c) and leatherbacks on the right column (b, d). Relationships with the fraction of time spent diving (%DT) are on the top line (a, b) and relationships with Maximum Daily Diving Depth (M3D) are on the bottom line (c, d). Confidence intervals are shown as grey shading and the black lines on the x-axis show the distribution of the observation data.
